# Pathomechanisms and Clinical Implications of Myasthenic Syndromes Exacerbated and Induced by Medical Treatments

**DOI:** 10.3389/fnmol.2020.00156

**Published:** 2020-08-14

**Authors:** Martin Krenn, Anna Grisold, Philipp Wohlfarth, Jakob Rath, Hakan Cetin, Inga Koneczny, Fritz Zimprich

**Affiliations:** ^1^Department of Neurology, Medical University of Vienna, Vienna, Austria; ^2^Division of Blood and Marrow Transplantation, Department of Medicine I, Medical University of Vienna, Vienna, Austria; ^3^Division of Neuropathology and Neurochemistry, Department of Neurology, Medical University of Vienna, Vienna, Austria

**Keywords:** drug-related myasthenia, neuromuscular transmission, neuromuscular junction, drug-induced myasthenia, immune checkpoint inhibitors

## Abstract

Myasthenic syndromes are typically characterized by muscle weakness and increased fatigability due to an impaired transmission at the neuromuscular junction (NMJ). Most cases are caused by acquired autoimmune conditions such as myasthenia gravis (MG), typically with antibodies against the acetylcholine receptor (AChR). Different drugs are among the major factors that may complicate pre-existing autoimmune myasthenic conditions by further impairing transmission at the NMJ. Some clinical observations are substantiated by experimental data, indicating that presynaptic, postsynaptic or more complex pathomechanisms at the NMJ may be involved, depending on the individual compound. Most robust data exist for the risks associated with some antibiotics (e.g., aminoglycosides, ketolides, fluoroquinolones) and cardiovascular medications (e.g., class Ia antiarrhythmics, beta blockers). Apart from primarily autoimmune-mediated disorders of the NMJ, *de novo* myasthenic manifestations may also be triggered by medical treatments that induce an autoimmune reaction. Most notably, there is growing evidence that the immune checkpoint inhibitors (ICI), a modern class of drugs to treat various malignancies, represent a relevant risk factor to develop severe and progressive medication-induced myasthenia via an immune-mediated mechanism. From a clinical perspective, it is of utmost importance for the treating physicians to be aware of such adverse treatment effects and their consequences. In this article, we aim to summarize existing evidence regarding the key molecular and immunological mechanisms as well as the clinical implications of medication-aggravated and medication-induced myasthenic syndromes.

## Introduction

Myasthenia gravis (MG) is the prototype disorder of the neuromuscular junction (NMJ) typically characterized by an autoimmune-mediated muscle weakness and premature fatigue. In more than 80% of patient sera, antibodies directed against the nicotinic acetylcholine receptor (AChR) can be detected (Cetin and Vincent, 2018; Koneczny and Herbst, 2019). Other conditions affecting neuromuscular transmission include Lambert-Eaton myasthenic syndrome (LEMS) (Titulaer et al., 2011), a range of congenital myasthenic syndromes (CMS) due to variants in > 30 different genes (e.g., *CHRNE*, *RAPSN*, *DOK7*, etc.) (Vanhaesebrouck and Beeson, 2019) and botulism (Guidon, 2019), all of which may be caused by various presynaptic, postsynaptic or combined mechanisms at the NMJ. In addition, drugs or medical treatments may also compromise neuromuscular transmission, the physiology of which will be introduced before outlining the mechanisms underlying the exacerbation of myasthenic syndromes by medical treatments.

Upon arrival of an action potential at the nerve terminal, presynaptic voltage-gated calcium channels (VGCC) open and give rise to an increase of the intracellular Ca^2+^ concentration. This triggers the fusion of presynaptic vesicles containing acetylcholine (ACh) with the cell membrane, releasing their content into the synaptic cleft. In humans, around 50 presynaptic vesicles are released upon each nerve action potential (i.e., quantal content). AChRs are densely concentrated at the postsynaptic muscle membrane by rapsyn, an intracellular 43 kDa receptor-associated protein of the synapse (Maimone and Merlie, 1993), and bind up to two ACh molecules and become permeable to cations with a net inflow of sodium. The activation of AChRs by the release of the quantal content leads to the endplate potential (EPP) at the postsynapse, a locally restricted depolarization of the muscle membrane. Occasional spontaneous release of a single vesicle gives rise to the much smaller (quantal) miniature endplate potential (MEPP). The EPP usually exceeds the threshold potential of –50 mV required for the activation of voltage-gated sodium channels (VGSC) at the depths of the postsynaptic folds, which triggers an action potential in the muscle fiber. The summation of all muscle fiber action potentials generated by the stimulation of a motor axon results in the compound muscle action potential. A safety factor defines the excess local depolarization of the muscle membrane that exceeds the threshold potential required for VGSC activation, ensuring that each nerve action potential is translated into a muscle action potential under physiological conditions. Different factors including the release of a substantially high number of presynaptic vesicles together with high AChR and VGSC densities at the postsynaptic membrane contribute to the safety factor that is around 2 in humans (Elmqvist et al., 1964; Wood and Slater, 2001). There is a non-linear relationship between the quantal content and the EPP. As the membrane potential approaches the reversal potential of the AChR (around 0 mV) during the EPP, the driving force for the cationic inward current through AChRs also decreases, so that with an increasing quantal content more quanta have to be released to cause a further unit increase in EPP amplitude (Slater, 2008).

**FIGURE 1:**
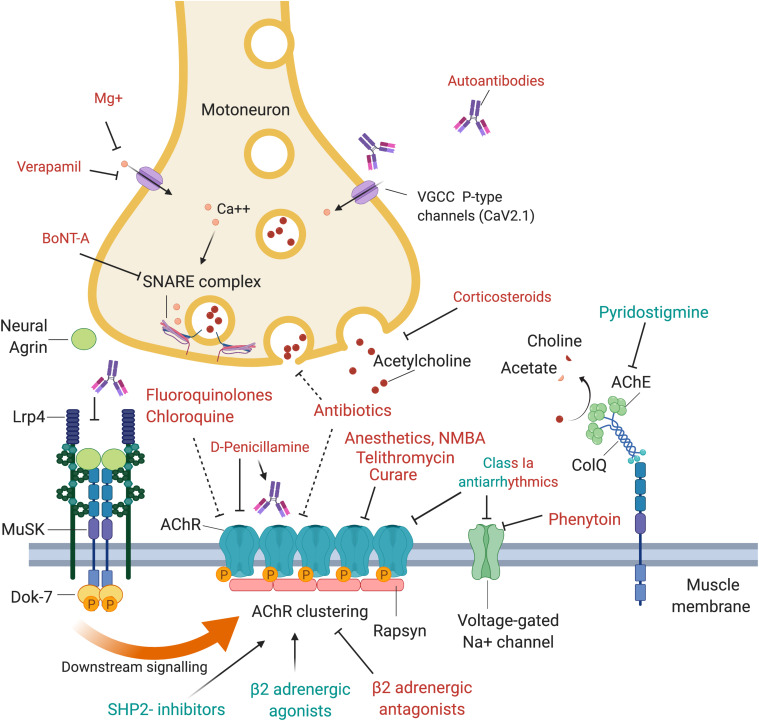
Drugs and antibodies that affect the function of the neuromuscular junction. Upon arrival of an action potential, presynaptic P-type VGCC (voltage-gated calcium channels) open and calcium enters the motoneuron terminal. This may be negatively affected by autoantibodies that block the VGCC, Mg^+^ or Verapamil. Calcium stimulates Ca^2+^ mediated exocytosis of neurotransmitters via the SNARE complex, which is negatively affected by BoNT-A which induces cleavage of one of its components, SNAP-25. Release of acetylcholine may also be reduced by corticosteroids or antibiotics. At the postsynaptic side, AChRs are densely aggregated, which is regulated via the agrin-Lrp4-MuSK-Dok-7 signaling pathway. Neural agrin is released by the motoneuron and binds to its co-receptor, Lrp4, located in the muscle membrane. Lrp4 and agrin bind as a dimer of dimers to the muscle-specific kinase (MuSK), a receptor tyrosine kinase that then phosphorylates itself, leading to the attachment of the adapter protein Dok-7. This binding leads to a full activation of MuSK and phosphorylation of downstream molecules, cumulating in the phosphorylation and dense aggregation of AChRs. The interaction between MuSK and Lrp4 can be blocked by autoantibodies, leading to interrupted signal transduction and reduced AChR clustering. The AChR clustering can be strengthened by SHP2-inhibitors and β2-adrenergic agonists, and de-stabilized by β2-adrenergic antagonists. Binding of acetylcholine to the AChR leads to opening of the channel, influx of cations, depolarization of the membrane and opening of voltage-gated Na + channels, which amplifies the depolarization. A range of drugs was shown to block the AChR, including fluoroquinolones, chloroquine, antibiotics, anesthetics, NMBA, telithromycin, curare and class Ia antiarrhythmics. Quinidine, a class Ia antiarrhythmic drug, may also be beneficial in the context of slow channel CMS with a pathological prolonged open state of the AChR. Voltage-gated Na^+^ channels are inhibited by phenytoin and class Ia antiarrhythmics. Acetylcholine is recycled in the synaptic cleft by the acetylcholinesterase (AChE), which hydrolyses acetylcholine to choline and acetate. AChE is arranged in tetramers attached to collagen q like tail (ColQ), which in turn is anchored to the synapse via binding to MuSK. AChE is inhibited by pyridostigmine, leading to prolonged presence of acetylcholine in the synaptic cleft, and is used as first line symptomatic treatment in myasthenia gravis.

Some medications, for instance various antibiotics or cardiovascular drugs (beta blockers, calcium channel blockers), that directly impair neuromuscular transmission due to different pre- or postsynaptic or combined mechanisms, usually cause transient worsening of symptoms in patients with a previously diagnosed myasthenic condition (Figure 1). It can be hypothesized that most of such drug-related adverse events that are caused by interference with the NMJ only last for a short period of time, mainly depending on the half-life of the drug and patient-related factors (gender, age, renal function, etc.) influencing its elimination. One of the few studies systematically addressing this issue reported that 19% of all recorded MG exacerbations were due to pharmaceutical interventions (Gummi et al., 2019). Withdrawal of myasthenia-aggravating drugs should be effective in these cases, and once an acute aggravation has been managed successfully, the clinical baseline may again be reached without further intervention.

**FIGURE 2:**
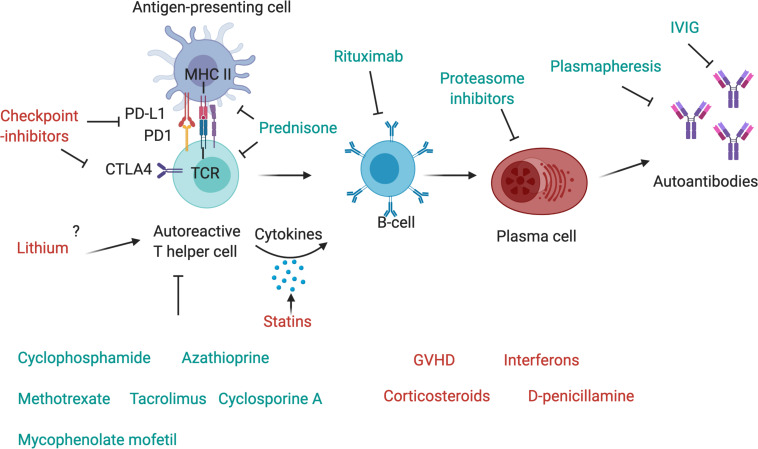
Drugs affecting components of the immune system. The T-cell receptor (TCR) of autoreactive T-helper cells recognizes antigen peptides that are presented by the MHC II complex on antigen-presenting cells. The binding of programmed cell death protein 1 (PD-1) to its ligand, PD-L1, expressed, respectively, on antigen-presenting cell and T-cell, inhibits immune activation and is an important immune checkpoint to guard against autoimmunity. CTLA4 is another checkpoint that is expressed on regulatory T-cells and upregulated on conventional T cells after activation and turns off T-cell activation when it interacts with its ligands, CD80 or CD86, on antigen-presenting cells. Checkpoint inhibitors are used as therapy to stimulate an attack on tumor cells, but they may also activate autoreactive T-cells and induce autoimmunity. Corticosteroids such as Prednisone inhibit antigen processing and presenting in antigen-presenting cells and block T-cell activation in the nucleus. T-cells are further targeted by other immunosuppressant drugs, such as cyclophosphamide, azathioprine, methotrexate, mycophenolate mofetil, cyclosporin A or tacrolimus. T helper cells activate autoreactive B-cells via MHC II-antigen-TCR interaction and the secretion of pro-inflammatory cytokines, these may be increased by statins. B-cells can be depleted by the use of Rituximab, a therapeutic monoclonal antibody against CD20, which is beneficial in many antibody-mediated autoimmune diseases. Plasma cells, which produce autoantibodies, may be targeted by proteasome inhibitors, and autoantibodies can be depleted by plasmapheresis. IVIg are also a therapeutic drug in autoimmune diseases that ameliorate antibody-mediated effects, although their exact mechanism of action is unknown.

In contrast to these directly NMJ-related mechanisms, a small number of drugs (e.g., D-penicillamine, immune checkpoint inhibitors) or treatments (e.g., allogeneic stem cell transplantation) may lead to *de novo* myasthenic syndromes (Figure 2). This is primarily explained by setting in train a previously unknown autoimmune process that subsequently affects neuromuscular transmission, similar to classical autoimmune myasthenia (Penn et al., 1998). In such cases, simply stopping the causative treatment may not be sufficient to reverse the symptoms right away, as enduring auto-reactive immune responses have been initiated. Based on this pathophysiological concept, it can be presumed that immunomodulatory treatments may be more effective in treating medication-induced myasthenic syndromes caused by immune-related mechanisms.

In this review article, we aim to summarize the well-characterized molecular mechanisms of various medical treatments related to worsening of myasthenia (summarized in Table 1) or the development of *de novo* myasthenic symptoms (summarized in Table 2). Since evidence is scanty for many classes of medication, we will also descriptively mention drugs/treatments with as yet unknown mechanisms but a potential clinical relevance.

**TABLE 1:** Selection of clinically relevant classes of drugs associated with exacerbations of pre-existing myasthenic syndromes.

Class of drugs	Main mode of action	Myasthenia-related pathomechanism(s)	Clinical features	Evidence supporting myasthenia-related effects	Additional information	References
Aminoglycoside antibiotics	Inhibition of protein synthesis	Pre-, postsynaptic, and combined (depending on compound)	Dose-dependent muscle weakness partially reversible by AChEI and calcium	Case reports, *in vitro* data, electrophysiological investigations	Neomycin most toxic, tobramycin least toxic	Pittinger and Adamson, 1972; Singh et al., 1978a, b; Caputy et al., 1981; Kaeser, 1984
Fluoroquinolone antibiotics	Gyrase inhibition	Postsynaptic blockade of AChRs	Rapid clinical worsening of known MG or unmasking MG	Large number of case-based evidence; chemical similarity to quinine, quinidine and chloroquine, which cause neuromuscular blockade	Levofloxacin, ofloxacin and ciprofloxacin cause severe exacerbations (FDA warning)	Moore et al., 1988; Mumford and Ginsberg, 1990; Azevedo et al., 1993; Roquer et al., 1996; De Sarro and De Sarro, 2001; Gunduz et al., 2006
Macrolide/ketolide antibiotics (telithromycin)	Interference with protein synthesis via ribosomal 50S subunit	Postsynaptic blockade of AChRs	Symptom aggravation within 2 h after first telithromycin administration	Case series with 10 patients, *in vitro* data (whole-cell patch-clamp)	Telithromycin withdrawn from market	May and Calvert, 1990; Cadisch et al., 1996; Nieman et al., 2003; Jennett et al., 2006; Perrot et al., 2006; Liu and Somps, 2010
Class Ia antiarrhythmics (procainamide, quinidine)	Blockade of sodium channels	Pre- and postsynaptic blockade	Procainamide – rapid and severe deterioration of weakness in MG; Quinidine – potential to unmask MG	Microelectrode and patch clamp recordings, animal models (rodent)	Quinidine used as a treatment in (slow channel) CMS	Lee et al., 1983; Oh et al., 1986; Godley et al., 1990; Yeh et al., 1992; Miller et al., 1993
Class IV antiarrhythmics (verapamil)	Blockade of voltage-dependent Ca^++^ channels	Presynaptic reduction of released ACh (but also postsynaptic effects?)	Clinical worsening of LEMS and MG	Single case-based observations, *in vitro* data, electrophysiological investigations	–	Krendel and Hopkins, 1986; Ribera and Nastuk, 1989; Swash and Ingram, 1992; Jonkers et al., 1996; Protti et al., 1996
Beta-adrenergic blockers	Selective or non-selective blockade of beta-adrenergic receptors	Destabilizing effect on AChR clusters	Worsening of previously diagnosed as well as newly diagnosed MG	Retrospective chart review study, experimental data on inverse effects of beta agonists (for CMS)	2.7-fold increased risk of developing an MG aggravation	Harry et al., 1974; Verkijk, 1985; Leys et al., 1987; Confavreux et al., 1990; Clausen et al., 2018; Gummi et al., 2019
Botulinum toxin A	Presynaptic blockade of ACh release	Presynaptic blockade of ACh release	Local and distant neuromuscular effects (flaccid weakness), mimicking or unmasking/aggravating MG, including dysphagia, ptosis, diplopia, limb weakness (latency: 1 day and 6 weeks)	Case reports, single-fiber electromyography (impaired neuromuscular transmission distant from injection site)	Unmasking of LEMS reported in 1 case; BoNT-A should be considered contraindicated in NMJ-related disorders	Erbguth et al., 1993; Garner et al., 1993; Watts et al., 2015; Dashtipour and Pedouim, 2016; Timmermans et al., 2019
Corticosteroids	Complex metabolic, anti-inflammatory, immunosuppressive, anti-proliferative effects	Altered depolarization of nerve cells, reduction of ACh release, changes in choline transport, depletion of potassium	Initial exacerbation, 8.6% severe, requiring mechanical ventilation or intubation	Case reports, observational study, experimental models	Gradually increasing steroid dose may avoid initial exacerbations	Seybold and Drachman, 1974; Wilson et al., 1974; Abramsky et al., 1975; Dengler et al., 1979; Kim et al., 1979; Pascuzzi et al., 1984; Kanai et al., 2019
Magnesium	Electrolyte with multiple metabolic functions, involved in hormone receptor binding, muscle contraction, neural activity, transmitter release, cardiac excitability	Reduced AChR release due to competitive block of calcium entry into the presynaptic nerve terminal; Possibly additional mild postsynaptic effect	Reminiscent of LEMS, ocular muscles tend to be spared	Electrophysiological investigations of nerve and muscle tissue	Parenteral use should be avoided in MG patients	del Castillo and Engbaek, 1954; Lipsitz, 1971; Gutmann and Takamori, 1973; Cohen et al., 1976; Swift, 1979; Pritchard, 1979; Krendel, 1990; Singh et al., 2015
Lithium	Unknown	Enhancing T-cell activity; reducing AChRs at NMJ	Aggravations of MG and unmasking of autoimmune myasthenia	Case reports, functional evidence derived from studies in rodents	Clinically used as a mood stabilizer	Neil et al., 1976; Mizuno et al., 1982; Lipton, 1987; Pestronk and Drachman, 1987; Ronzière et al., 2000
Phenytoin	Blockade of voltage-gated sodium channels	Pre- and postsynaptic effects	In some published cases immediate amelioration of symptoms after PHT discontinuation	Few case reports, *in vitro* studies	Weak evidence also for other AEDs (CBZ, GBP)	Norris et al., 1964; Brumlik and Jacobs, 1974; Yaari et al., 1977
Neuromuscular blocking agents	Competitively blocking the binding of ACh to its receptors (non-depolarizing) or depolarizing the muscle fiber	Postsynaptic blockade	Prolonged NMJ blockade in MG and LEMS, with severe disease and higher doses of pyridostigmine correlating with increased sensitivity	Case reports	Sugammadex, has the potential to reverse the neuromuscular blockade of NMBAs	Nilsson and Meretoja, 1990; Schaller and Lewald, 2016
Volatile (inhalation) anesthetics	Inhibition of nicotinic AChR	Changing kinetics of postsynaptic conductance at synapses	Increased sensitivity of MG patients regarding neuromuscular side effects	Case-based observations, experimental data	Thorough pre- and postsurgical management of patients with NMJ disorders	Gage and Hamill, 1976; Nitahara et al., 2007

*Ach, Acetylcholine; AChEI, Acetylcholine esterase inhibitors; AChR, Acetylcholine receptor; AED, anti-epileptic drug; BoNT-A, Botulinum toxin A; CBZ, Carbamazepine; CMS, Congenital myasthenic syndrome; FDA, Food and Drug Administration; GBP, Gabapentin; LEMS, Lambert-Eaton myasthenic syndrome; MG, Myasthenia gravis; NMBA, Neuromuscular blocking agent; NMJ, neuromuscular junction; PHT, phenytoin.*

**TABLE 2:** Drugs and medical treatments with best evidence for an association with the development of *de novo* myasthenic syndromes.

Class of drugs/type of treatment	Main mode of action	Myasthenia-related pathomechanism(s)	Clinical features	Evidence supporting myasthenia-related effects	Additional information	References
D-penicillamine	Pyridoxine antagonist and chelating drug	Production of AChR Abs due to a newly developed immune response directed against the drug (and mild unspecific presynaptic effects)	Mostly mild ocular symptoms 2–12 months after initiation, usually responding well to pyridostigmine, remission often within 1 year after discontinuation	Large number of case reports, experimental immunological data	Used for the treatment of Wilson’s disease, MG phenotype observed in up to 7%	Vincent et al., 1978; Aldrich et al., 1979; Albers et al., 1981; Kuncl et al., 1986; Drosos et al., 1993; Andonopoulos et al., 1994; Adelman et al., 1995; Penn et al., 1998
Chloroquine	Anti-malarial drug that prevents biocrystallization of heme	Postsynaptic blockade of AChRs and additional immune-modulatory properties	Reminiscent of classical autoimmune MG	Case reports, electrophysiological investigations	Recently suggested as a COVID-19 drug, study reporting ineffectiveness has been retracted	Robberecht et al., 1989; Varan et al., 2015; Mehra et al., 2020
Interferon	Multiple effects including inhibition of protein synthesis, inactivation of viral RNA, phagocytic and cytotoxic mechanisms (leading to anti-viral, anti-tumor, anti-angiogenic activity)	Primarily humoral immune response, comparable to that in human MG	AChR-positive MG, often additional myopathic findings in EMG	Case reports for both inducing and aggravating myasthenia, transgenic mice studies, histological-immunological work-up	Most reported cases occurred when IFN was used for hepatitis C (additional pathogenic role of viral infection?)	Conlon et al., 1990; Batocchi et al., 1995; Gu et al., 1995; Borgia et al., 2001; Wolfe et al., 2007; Congeni and Kirkpatrick, 2013; Baik et al., 2016; Saleem, 2016
Allogeneic hematopoietic stem cell transplantation	Transfer of the donor hematopoietic and immune system to the host to treat (hematological malignancies)	Combined cellular (T- and B-cells) and humoral alloreactivity as a manifestation of chronic GVHD	Develops 3–12 months after HSCT, often supported by AChR Abs	Large-scale observational data	Rare occurrence with incidence < 1%	Bolger et al., 1986; Lefvert and Björkholm, 1987; Maffini et al., 2017; Tsutsumi et al., 2019
Immune checkpoint inhibitors	Inhibition of checkpoint molecules CTLA4, PD-1, PD-L1	Induction of autoimmune activity due to interference with immunological homeostasis, reduced T-cell tolerance, increase of inflammatory cytokines	Very severe and progressive clinical course, often accompanied by respiratory failure, overall mortality 20–30%; AChR Abs in 66%; overlap syndromes with myositis/myocarditis	Large-scale pharmacovigilance studies, numerous case reports/series	Incidence approximately 0.5%; Most commonly due to PD-1 or PD-L1 inhibition	Johnson et al., 2015, 2019; Montes et al., 2018; Sato et al., 2019; Safa et al., 2019; Xing et al., 2020
Statins	HMG-CoA reductase inhibitors	Increase of Th2 interleukins, depletion of coenzyme Q10	Predominantly affecting oculo-bulbar muscles with symptom onset between 1 week and 4 months after initiating treatment	Case series	Both aggravating and developing myasthenia has been described	Parmar et al., 2002; Cartwright et al., 2004; Purvin et al., 2006; Oh et al., 2008; Khalid et al., 2016

*Ach, Acetylcholine; AChR, Acetylcholine receptor; COVID-19, coronavirus disease 2019; CTLA4, cytotoxic T-lymphocyte-associated protein 4; EMG, Electromyography; GVHD, Graft-versus-host disease; HSCT, Hematopoietic stem cell transplantation; HMG-CoA reductase, 3-hydroxy-3-methyl-glutaryl-coenzyme A reductase; IFN, Interferon; MG, Myasthenia gravis; PD-1, Programmed cell death protein 1; PD-L1, Programmed cell death 1 ligand 1; Th2, T helper type 2.*

A broad range of drugs have been reported to worsen neuromuscular weakness in patients with pre-existing NMJ disorders. The most commonly observed clinical effect is a transient aggravation of symptoms and a relatively prompt recovery upon drug withdrawal. However, for the same drugs also unmasking of a previously unknown myasthenic condition has been hypothesized, but this phenomenon is apparently difficult to prove based on case description. Generally speaking, these drugs usually directly interfere with NMJ function due to different pre- or postsynaptic and combined pathomechanisms.

## Presynaptic Neuromuscular Junction Effects

### The Botulinum Toxin Effect

One of the best-characterized and long-known mechanisms is the one caused by botulinum toxin A (BoNT-A), a toxin produced by *Clostridium botulinum*. BoNT-A is routinely used for the medical treatment of various (focal) neurological disorders (e.g., dystonia, spasticity). The main mode of action is the blockade of ACh release at the presynaptic nerve terminal. More specifically, BoNT-A causes proteolytic cleavage of SNAP-25, which is part of the soluble N-ethylmaleimide-sensitive-factor attachment receptor (SNARE) complex and crucially involved in neurotransmitter release through Ca^2+^-mediated exocytosis (Dolly and Aoki, 2006).

Although transient and usually mild, typical side effects include neuromuscular symptoms through local (to the injection site) but also distant effects at the NMJ, including dysphagia, ptosis, diplopia and limb weakness (Dashtipour and Pedouim, 2016). Interestingly, single-fiber electromyography demonstrated changes in neuromuscular transmission in muscles distant from the injection site (Garner et al., 1993). In keeping with these observations, myasthenic disorders may significantly worsen due to BoNT-A (Watts et al., 2015). As a result, pre-existing disorders of the NMJ represent a relative contraindication for the injection of BoNT-A, hence requiring a cautious risk-benefit assessment by the treating clinicians beforehand.

In addition to the known NMJ-related side effects, anecdotal evidence suggests an association between BoNT-A injections and an initial manifestation of a myasthenic condition. To the best of our knowledge, eight cases have so far reported a first manifestation of MG following BoNT-A injection, with the majority of described patients showing predominantly ocular symptoms with a latency between 1 day and 6 weeks after injection (Timmermans et al., 2019). Moreover, one publication reported that LEMS first presented after BoNT-A injection (Erbguth et al., 1993). Given the known directly NMJ-related mechanism, it can be assumed that previously subtle conditions may have been unmasked by further impairing neuromuscular transmission.

### Electrolyte-Related Neuromuscular Junction Effects

An electrolyte-related impairment of NMJ function may be caused by electrolyte imbalances, most typically by an excess of magnesium. Magnesium competitively blocks the entry of calcium into the presynaptic nerve terminal – a process that is crucial for the release of ACh into the synaptic cleft. The subsequent magnesium-induced reduction of ACh release eventually impairs neuromuscular transmission (Krendel, 1990). Accordingly, the pathophysiological mechanisms of hypermagnesemia resemble LEMS, in which Abs are directed against the presynaptic VGCC. Investigations of nerve and muscle tissue showed that endplate potential amplitudes evoked by nerve stimulation were reduced in the presence of magnesium, which could be reversed by adding calcium. This finding corroborates a presynaptic mechanism as the key mode of action. A slight decrease in sensitivity of the motor endplate to ACh was also observed, potentially suggesting a mild additional postsynaptic effect (del Castillo and Engbaek, 1954).

In concordance with the mainly presynaptic mode of action, the clinical picture also resembles LEMS rather than classical MG (Swift, 1979). Nonetheless, also patients with known disorders of the postsynaptic membrane (i.e., MG) appear to react particularly sensitive to magnesium. Hence, individuals with MG and LEMS may develop increased weakness already at slightly elevated serum levels (Gutmann and Takamori, 1973; Cohen et al., 1976).

An excess of magnesium may especially be relevant in patients with concomitant renal failure and the use of magnesium-containing drugs such as laxatives, antacids or enemas (Krendel, 1990). Moreover, hypermagnesemia is a pharmacologically intended state in women with (pre)eclampsia (Pritchard, 1979), and the application of high parental doses may result in myasthenic crises in these patients (Lipsitz, 1971; Singh et al., 2015). Clinical symptoms have been reported to correlate well with magnesium serum levels (Fishman, 1965). In patients with no previous history of neuromuscular disease but myasthenic symptoms due to iatrogenic magnesium application, magnesium withdrawal and the intravenous use of calcium can be efficient. Hemodialysis may remain reserved for severe cases (Alfrey et al., 1970). In contrast, low oral doses of magnesium are usually not considered as a clinically relevant problem.

### Calcium-Dependent Presynaptic Effects

As mentioned above, the influx of calcium through VGCC, induced by a motor axonal action potential, represents one of the key functional events of neuromuscular transmission in the presynaptic nerve terminal. Yet, the exact influences of calcium channel blocking agents (which are mainly used for cardiovascular indications) on neuromuscular activity are not fully understood, but some functional data and clinical reports suggest a clinically relevant impairment of neuromuscular function.

From an experimental point of view, a presynaptic reduction of released ACh but also postsynaptic effects have been observed for the calcium channel blocker verapamil (Ribera and Nastuk, 1989). When studying the effects of different calcium channel blockers on transmitter release at the neuromuscular junction, it was found that P-type calcium channel blockers inhibited nerve-evoked action potentials and subsequent synaptic transmission, while transmitter release remained unaffected by selective L-type and N-type channel blocking agents (Protti et al., 1996). These findings underscore that P-type channels (Ca_V_2.1) are the primary mediators of transmitter release at motor nerve terminals.

In addition, case-based observations of paraneoplastic LEMS and autoimmune MG showed clinical worsening following a treatment with verapamil, a class IV antiarrhythmic drug that blocks voltage-dependent calcium channels (Krendel and Hopkins, 1986; Swash and Ingram, 1992). The effect of verapamil on neuromuscular transmission was addressed by Jonkers et al., who studied the influence of intravenous verapamil on repetitive nerve stimulation and clinical function in 10 patients with MG. However, no reproducible effects on neuromuscular transmission were found in this study, that was mainly limited by its small sample size (Jonkers et al., 1996).

## Postsynaptic Neuromuscular Junction Effects

### Blockade of Acetylcholine Receptors (Curare-Like Effects)

The plant-derived toxin curare is a historical example for neuromuscular blockade, acting by a competitive inhibition of the nicotinic AChR at the postsynaptic membrane. Functionally similar (curare-like) mechanisms have also been proposed for various drugs.

A broad range of antibiotics have already been associated with clinical worsening in patients with MG. This is particularly relevant, since many patients with MG are under immunosuppressive treatment, partly suffering from recurrent respiratory symptoms and requiring antimicrobial treatment. Yet, the exact mechanisms underlying neuromuscular blockade are diverse and often poorly understood.

First, Telithromycin, a relatively new ketolide antibiotic, is known to induce myasthenia or lead to severe symptom exacerbations including fatal respiratory failure and death (Nieman et al., 2003; Jennett et al., 2006). *In vitro* studies substantiated these observations by showing that telithromycin inhibits nicotinic AChRs and blocks neuromuscular transmission (Liu and Somps, 2010). A case series comprising 10 affected individuals highlights the potential to aggravate and unmask MG, also reporting that most patients develop symptoms within 2 h after the first administration of telithromycin, which is in line with the aforementioned mechanism (Perrot et al., 2006). The drug has already been withdrawn from the market due to its severe side effects.

There is a long list of anecdotal reports indicating (sometimes severe) symptom deterioration following the administration of fluoroquinolones, i.e., gyrase inhibitors, which led to a “black box” warning by the FDA, for their use in patients with MG (De Sarro and De Sarro, 2001). Notably, the fluoroquinolones, particularly levofloxacin, ofloxacin and ciprofloxacin, may cause acute and severe exacerbations of myasthenic symptoms (Moore et al., 1988; Mumford and Ginsberg, 1990; Azevedo et al., 1993; Roquer et al., 1996; Gunduz et al., 2006). From a biochemical perspective, these adverse effects may in part be explained by the structural similarities to quinine and chloroquine, which have been shown to cause a neuromuscular blockade (Sieb et al., 1996).

Chloroquine is used for the treatment of malaria, but also for some rheumatological diseases. Moreover, the pandemic COVID-19 caused by the virus infection with SARS-CoV-2 has recently brought widespread attention to this drug, as it has initially been suggested as a potentially promising anti-viral treatment for this indication (Touret and de Lamballerie, 2020). A subsequent multi-national analysis showing an increased mortality rate and no beneficial effects was later retracted by the journal (Mehra et al., 2020). In general, clinical, electrophysiological and serological characteristics of chloroquine-related myasthenia are strikingly reminiscent of acquired autoimmune MG. In view of the complete recovery 2 weeks after treatment cessation (compatible with the long half-life of chloroquine) and the absence of anti-AChR Abs, it has been hypothesized that chloroquine-induced MG may be caused by a direct postsynaptic mechanism (Robberecht et al., 1989). However, in contrast to most classical antimicrobial drugs, chloroquine seems to have the ability to induce (partly anti-AChR-positive) MG, usually recovering after drug withdrawal (Varan et al., 2015). It is worthy of note, that chloroquine has immune-modulatory properties that may play an additional role in such cases.

Primarily postsynaptic effects at the NMJ have also been ascribed to class Ia antiarrhythmics, which interfere with sodium channels. Intracellular microelectrode recordings in rats provided evidence for both pre- and postsynaptic effects of procainamide, with postsynaptic blockade representing the dominant mode of action (Lee et al., 1983). One study by Yeh et al. (1992) demonstrated a perturbed AChR-dependent contractile muscle function in a rat model of experimental MG caused by procainamide. Clinically, procainamide may lead to a rapid and severe deterioration of weakness in MG patients (Godley et al., 1990). Moreover, also the primary induction of myasthenic symptoms including bulbar features has been reported occasionally in the literature (Oh et al., 1986; Miller et al., 1993). Though immunological properties have been ascribed to this drug, the rapid clinical manifestation of neuromuscular symptoms rather supports a directly NMJ-related mechanism.

Drug-induced side effects that might be related to alterations of the membrane potential of the NMJ are most obvious for the neuromuscular blocking agents (NMBAs), which are used in anesthesiology. In fact, both depolarizing and non-depolarizing NMBAs may interfere with the muscle membrane potential. Relatively small doses of non-depolarizing agents can cause a profound and prolonged NMJ blockade in individuals with MG and LEMS, with more severe disease and higher doses of pyridostigmine correlating with an increased sensitivity toward non-depolarizing NMBAs (Nilsson and Meretoja, 1990). A comparably novel drug, sugammadex, has the potential to reverse the neuromuscular blockade of NMBAs within minutes by 1:1 binding of rocuronium or vecuronium (Schaller and Lewald, 2016). Given most reports published to date, it can be considered as a safe option to restore neuromuscular transmission for this vulnerable patients population, if the use of NMBAs is necessary (Cata et al., 2019).

It is a commonly observed phenomenon that patients with NMJ disorders are specifically prone to prolonged weakness after surgery with various types of anesthesia. Patients with MG pose a major challenge for anesthesiologists, and the post-surgical risk of respiratory problems is a matter of concern. Yet, surgery is often necessary in MG, especially because thymectomy is a standard treatment of seropositive patients with AChR Abs (Wolfe et al., 2016). A careful selection of anesthetic drugs must be made to avoid complications. Elective surgery should ideally be performed in a stable phase of myasthenic disease, and the clinical situation should be optimized beforehand (Jamal and Herb, 2009).

may result in a superimposed impairment of neuromuscular transmission when used together with NMBAs, volatile (inhalation) anesthetics may interfere with neuromuscular transmission in a more direct way, that is mainly through the inhibition of nicotinic AChR. Experimental data indicate that they change the kinetics of postsynaptic conductance at synapses (Gage and Hamill, 1976). Myasthenic patients display an increased sensitivity to the relaxant effects of these drugs (e.g., sevoflurane) (Nitahara et al., 2007). Regarding anesthesia, a thorough pre- and post-operative evaluation, the continuation of pyridostigmine and a careful monitoring should be helpful to safely manage patients with MG. In addition, it is important to use neuromuscular monitoring (i.e., train-of-four monitoring) during surgery to ensure optimal recovery before terminating anesthesia (Blichfeldt-Lauridsen and Hansen, 2012).

### Effects of Beta-Adrenergic Blockade

Beta-adrenergic blocking agents are under the suspicion of adversely affecting neuromuscular strength in MG patients (Verkijk, 1985). *In vitro* studies of nerve-muscle preparations revealed that various beta-adrenergic blockers may lead to a dose-dependent reduction of neuromuscular transmission in rats with propranolol having the most pronounced effect on neuromuscular transmission (Harry et al., 1974). Adrenergic beta-2 receptor agonists are increasingly used in the treatment of CMS, and it has been shown that they directly interfere with proteins located at the NMJ, exerting a stabilizing effect on AChR clusters (Clausen et al., 2018). This strongly suggests a reverse effect at the NMJ as the mechanism underlying beta blocker-related worsening of myasthenic symptoms. It is also worthy of note that the stabilization of AChR clusters using an SRC homology 2 domain-containing phosphotyrosine phosphatase 2 (SHP2) inhibitor has recently been found in early cell culture studies to be a promising mechanisms against MuSK-MG (Huda et al., 2020).

A retrospective chart review study showed that the odds of developing a transient aggravation of MG is increased 2.7-fold under beta-adrenergic antagonists in general, which represents a statistically significant signal (Gummi et al., 2019). A small-scale systematic trial studying the neuromuscular effects of an intravenously applied beta blocker (propranolol) in 10 patients could not prove an acute and significant deterioration of neuromuscular transmission in MG using repetitive nerve stimulation and clinical tests, but the work was clearly limited by the small number of participants (Jonkers et al., 1996). Several case reports indicated that latent myasthenia may be unmasked by beta blocker treatment (Leys et al., 1987; Confavreux et al., 1990).

Apart from cardiovascular indications, beta-adrenergic antagonists are used for the local treatment of open angle glaucoma to depress intraocular pressure. Although applied as eye drops, there may be systemic adverse events including increased neuromuscular weakness in rare cases. Both worsening of previously diagnosed as well as newly diagnosed MG have been described for the non-selective beta blocker timolol (Coppeto, 1984; Verkijk, 1985) and for the beta1-selective blocker betaxolol (Khella and Kozart, 1997).

## Presumed (Combined) Pre- and Postsynaptic Effects

In many classes of drugs, the functional evidence for the pathomechanisms underlying neuromuscular blockade is scanty at best. Often, both pre- and postsynaptic mechanisms are presumed, mostly on the basis of electrophysiological studies. Hence, for many of these medications, the exact mechanism remains unknown.

Various reports have been published for aminoglycoside antibiotics to cause a clinically relevant neuromuscular blockade. Functional data indicate that aminoglycoside antibiotics may impair neuromuscular transmission through both presynaptic (via inhibition of prejunctional ACh release) and postsynaptic (via depression of post-junctional sensitivity) molecular mechanisms, which are overall still poorly appreciated (Pittinger and Adamson, 1972). The resulting effects appear to be (partly) reversible by calcium gluconate, AChE inhibitors and aminopyridines (Singh et al., 1978a, b). One study evaluated neuromuscular toxicity of several aminoglycosides including amikacin, gentamicin, kanamycin, neomycin, netilmicin, streptomycin and tobramycin, with neomycin being classified as the most toxic and tobramycin as the least toxic of all investigated aminoglycosides (Caputy et al., 1981). Interestingly, gentamicin, neomycin, streptomycin, tobramycin, and kanamycin have also been implicated in clinical weakness occurring in otherwise healthy (non-myasthenic) individuals (Kaeser, 1984).

Neuromuscular blockade due to antibiotics is certainly not confined to aminoglycosides. The monobasic amino acid antibiotics lincomycin and clindamycin structurally differ from classical aminoglycosides, but may cause a neuromuscular blockade which can be reversed by calcium and aminopyridines (Booij et al., 1978). Electrophysiological studies showed that both drugs cause the blockade via different modes of action. Lincomycin displayed predominantly nerve-terminal depressant properties, while clindamycin showed stimulatory presynaptic effects (Rubbo et al., 1977). However, real-life data confirming the clinical relevance of these *in vitro* effects are still lacking.

Polymyxin B and colistin, which are used as a last-resort for resistant Gram-negative bacteria, show neuromuscular toxicity, mainly in patients with renal failure or in combination with other NMJ-blocking drugs, perhaps potentiating their effects (Pittinger and Adamson, 1972). Again, the presumed mechanisms of action are a reduced release of ACh and, to a certain degree, the concomitant postsynaptic blockade of AChR receptors (Wright and Collier, 1976; Viswanath and Jenkins, 1978; Durant and Lambert, 1981).

Aside from antimicrobial drugs, the class Ia antiarrhythmic drug quinidine (and its stereoisomer quinine) have been observed to transiently worsen neuromuscular weakness in patients with MG (Weisman, 1949). Somewhat paradoxically at first sight, while deteriorating MG, quinidine represents an effective therapeutic option in (slow channel) CMS (Lee et al., 2018). In such cases with a pathologically prolonged open state of the AChR channel due to monogenic mutations, an open-channel blocker can be of clinical benefit (Peyer et al., 2013). The adverse effects causing neuromuscular blockade are localized in the presynaptic membrane, impairing ACh release, but also (to a lesser extent) in the postsynaptic membrane with curare-like effects, as demonstrated by microelectrode and patch-clamp investigations (Sieb et al., 1996).

One anti-epileptic drug (AED) that has been associated with myasthenic symptoms a long time ago is phenytoin (PHT), a blocker of voltage-gated sodium channels. *In vitro* studies demonstrated both presynaptic and postsynaptic effects on neuromuscular transmission (Yaari et al., 1977). Aside from functional data, few reports with an MG-like presentation have been published in the literature, often ameliorating after PHT discontinuation, potentially pointing toward an actual drug-associated effect (Norris et al., 1964; Brumlik and Jacobs, 1974).

## Immunological Mechanisms in Drug-Related Myasthenia

As opposed to the delineated NMJ-related drug effects, some medical treatments are known or presumed to set in motion a *de novo* immunological process, thus initiating new-onset autoimmune phenomena that may clinically resemble acquired conditions such as MG. This pathophysiological concept is in line with new-onset diseases in previously healthy individuals rather than a transient aggravation of a pre-existing disorder. However, also previous autoimmunological conditions may be more likely to flare up as a consequence of immune-modulatory treatments.

### D-Penicillamine

Occurring in up to 7% of patients taking D-penicillamine (D-P), myasthenia represents a relatively common adverse event (Andonopoulos et al., 1994). Clinical, electrophysiological and serological features do not seem to differ remarkably from classical MG. Most patients present with mild, predominantly ocular symptoms occurring 2–12 months after D-P initiation and usually responding to pyridostigmine (Drosos et al., 1993). However, marked respiratory symptoms have also been reported (Adelman et al., 1995). In many cases, D-P-related MG remits within 1 year following drug discontinuation (Albers et al., 1981). The prolonged latency of symptom onset and remissions and the gradual decline of Ab levels after withdrawal are in keeping with the mechanism of a drug-induced immunological effect (Vincent et al., 1978).

Basic research also supports that the production of AChR Abs plays an essential role and that D-P-induced MG may arise due to a newly developed immune-mediated response that is directed against the compound itself (Penn et al., 1998). Studies of D-P reacting with purified AChR demonstrated a covalent attachment to two receptor subunits (alpha and gamma). Moreover, D-P appears to impact the equilibrium of ACh binding properties of both purified receptor and receptor-rich membrane fragments (Bever et al., 1982). The detailed characterization of a patient with D-P-induced myasthenia indicated pathophysiological overlaps with idiopathic MG, including the production of anti-AChR Abs, as well as subsequent degradation and quantitative reduction of junctional AChRs (Kuncl et al., 1986). Electrophysiological changes in rats indicated also mild and unspecific presynaptic effects of D-P at high concentrations (Aldrich et al., 1979). Of note, the lower incidence of D-P-induced myasthenia in patients with Wilson’s disease compared to those with rheumatoid arthritis may suggest a general immunogenetic susceptibility to autoimmune disorders as an additional role (Komal Kumar et al., 2004).

### Interferon

Similar to D-P, patients under interferon (IFN) treatment may develop autoimmune-mediated disorders for the first time (Conlon et al., 1990). Among others, generalized, partly severe manifestations of MG were either triggered or aggravated by IFN alpha (Borgia et al., 2001; Wolfe et al., 2007; Congeni and Kirkpatrick, 2013; Baik et al., 2016; Saleem, 2016).

Studies in transgenic mice confirmed that the presence of IFN gamma in the NMJ results in generalized flaccid weakness and disrupted neuromuscular transmission that could successfully be reversed by AChE inhibitors (AChEi) such as pyridostigmine. The histological work-up identified mononuclear cells and Ab deposition at motor end plates. Immunoprecipitation found a novel target antigen which was recognized by sera from investigated mice but also from human MG patients. These data associate IFN with a humoral immune response, comparable to that in human MG pathogenesis (Gu et al., 1995).

Clinically, reported cases are often accompanied by the presence of AChR Abs and sometimes by additional myopathic findings on electromyography (Batocchi et al., 1995). MG was found to occur under a treatment with IFN beta for multiple sclerosis in three unrelated cases, in all of which it manifested within the first year after initiation (Blake and Murphy, 1997; Dionisiotis et al., 2004). Despite the lack of systematic data, the pathophysiological rationale as well as the time span from drug initiation to symptom onset support immune-related rather than NMJ-related effects.

### Statins

Statins, the most commonly used class of lipid-lowering drugs, also show a variety of immunomodulatory properties such as increasing the serum levels of multiple Th2 interleukins (Youssef et al., 2002). Well-fitting, studies in animals and humans indicate that Th2 cytokines may generally be crucial in the development of MG (Milani et al., 2003). Therefore, these changes may be responsible for inducing *de novo* MG (Gale and Danesh-Meyer, 2014). Another pathomechanism potentially underlying statin-induced myasthenia is the depletion of coenzyme Q_10_, which may result in mitochondrial dysfunction (Evans and Rees, 2002; Vaklavas et al., 2009). This is particularly relevant, as presynaptic endings and the postsynaptic junction have a high density of mitochondria.

In contrast to new-onset MG, non-inflammatory myopathy is a well-known side effect of statins, potentially explaining the case reports that suggest an aggravation of pre-existing MG (Parmar et al., 2002; Cartwright et al., 2004; Purvin et al., 2006). A small case series reported worsening of symptoms in 6 patients with MG receiving statins (11% of the small analyzed cohort), predominantly affecting oculo-bulbar muscles with symptom onset between 1 week and 4 months after initiating treatment (Oh et al., 2008). This latency appears too long for a mechanism directly related to NMJ function. Some reported patients had no previous history of myasthenic symptoms, indicating that statins may have the potential to trigger or unmask myasthenia in previously healthy individuals (Purvin et al., 2006; Khalid et al., 2016).

### Immune-Checkpoint Inhibitors

Immune checkpoint inhibitors (ICIs) are an emerging type of cancer immunotherapy that target intrinsic down-regulators of immunity, more specifically the checkpoint molecules CTLA-4, PD-1, and PD-L1 (Postow et al., 2018). These proteins may allow the proliferation of malignant cells by evading T-cell-mediated anti-tumor activity, and their blockade in turn enables T-cells to recognize and attack the tumor (Fife and Bluestone, 2008).

The checkpoint molecules, expressed on the surface of T-cells, play a crucial role in the regulation of immunological homeostasis, maintenance of self-tolerance and prevention of autoimmunity (Postow et al., 2015). Interference with immunological homeostasis and a reduction of T-cell tolerance are also postulated as crucial mechanisms in immune-related adverse events (Postow et al., 2018). ICIs may elevate the levels of pre-existing auto-Abs, but can also increase the levels of inflammatory cytokines, which may promote the activation of auto-reactive T cells (Kimbara et al., 2018). Additionally, the similarity between normal tissue antigens and tumor antigens may lead to a cross-reactivity, further favoring a self-directed immune response (Michot et al., 2016). Given these effects, activated T-cells may subsequently attack healthy tissue including structures of the NMJ, resulting in adverse reactions with clinical and serological features reminiscent of autoimmune diseases (Choi and Lee, 2020).

The clinical spectrum of immune-related adverse events includes a wide range of neurological complications affecting both the central and the peripheral nervous system (Bruna et al., 2020; Haugh et al., 2020). Together with peripheral neuropathies and myositis, MG is a characteristic side effect involving the peripheral nervous system, occurring in approximately 0.5% of cases receiving ICI treatment (Johnson et al., 2019). The median time period from drug administration to the clinical onset of MG was 28 days (Sato et al., 2019). ICI-triggered MG is an often severe and progressive complication with potentially life-threatening consequences and a mortality rate of up to 30% (Johnson et al., 2019; Safa et al., 2019). In the vast majority of reported cases with ICI-related MG, the underlying treatment was a pharmacological inhibition of PD-1 or its ligand PD-L1, most commonly with the drug nivolumab. Less commonly, cases under ipilimumab (targeting CTLA-4) have been described, and combination treatments seem to be particularly hazardous (Johnson et al., 2015; Montes et al., 2018).

In the single-center study by Safa et al., most patients (63%) developed moderate to severe disease (MGFA class III to V), with ptosis (75%), dyspnea (62%), generalized weakness (55%), dysphagia (48%), and double vision (42%) being the most commonly reported symptoms. Of note, almost half of the patients in this study rapidly developed respiratory failure requiring mechanical ventilation. Characteristic laboratory features were positive AChR Abs in 66%, anti-striated Abs in 67% and elevated CK levels in 84% (Safa et al., 2019). To the best of our knowledge, auto-Abs against the muscle-specific kinase (MuSK) have so far only been reported in one single case under nivolumab (occurring together with AChR Abs) (Mitsune et al., 2018).

ICI-induced MG may further be complicated by overlap manifestations comprising myositis or myocarditis with potentially fatal consequences (Xing et al., 2020). Overall, when compared to classic acquired MG, the clinical course of ICI-related disease clearly appears to be remarkably more aggressive with a tendency toward fast disease progression and myasthenic crises. Altogether, no significant clinical differences were found between patients with new-onset MG or an acute flare-up of pre-existing disease due to ICI (Safa et al., 2019).

Steroids are often used as first-line treatment approach, followed by immune-modulatory agents in refractory cases (Choi and Lee, 2020). However, the data presented by Safa et al. support the early use of intravenous immunoglobulins (IVIg) and plasma exchange (PLEX), leading to improved outcomes compared to steroids alone (Safa et al., 2019). The response to a symptomatic treatment with cholinesterase inhibitors seems to be variable (Johansen et al., 2019). As it is still not entirely clear whether ICI-induced MG is a monophasic disease, the decision regarding a possible withdrawal of ICIs remains a dilemma and should especially be discussed in life-threatening cases (Shi et al., 2020).

### Allogeneic Hematopoietic Stem Cell Transplantation

Allogeneic HSCT is a standard treatment for a variety of hematological malignancies involving the transfer of a donor’s hematopoietic and immune system to a host. Graft-versus-host disease (GVHD) is one of the major complications of the procedure and summarizes host end-organ damage due to alloreactivity of the donor’s immune system. T-cell infiltration and epithelial damage to the skin, gut and liver are the hallmarks of acute GVHD, usually occurring within the first 100 days after HSCT (Zeiser and Blazar, 2018). Chronic GVHD typically develops 3–12 months after HSCT and resembles a spectrum of autoimmune-related diseases through combined pathomechanisms of cellular (T- and B-cells) and humoral alloreactivity (Zeiser and Blazar, 2017).

Myasthenia-like manifestations have been described as rare immune-mediated complications after HSCT in case reports with an estimated incidence under 1%. The time of disease onset ranged between 3 and 100 months after HSCT, and a clear association with a prior diagnosis of chronic GVHD could be noted (Tsutsumi et al., 2019). In this context, MG has also been described during the taper of immunosuppressive medication used for the treatment of GVHD (Bolger et al., 1986). The detection of serum AChR Abs strongly supports the diagnosis of MG in the presence of classic symptoms after HSCT, but these auto-Abs can also be detected in up to 40% of HSCT recipients without apparent neurological symptoms (Lefvert and Björkholm, 1987). In a review of 27 post-HSCT cases with MG, treatment mainly consisted of prednisone in combination with a calcineurin inhibitor, azathioprine, IVIg and the anti-CD20 antibody rituximab. These treatments led to a sustained improvement in the majority of cases and only three deaths have been reported (Maffini et al., 2017).

Importantly, MG has to be distinguished from other causes of muscle weakness related to HSCT, including steroid myopathy, wasting syndrome or other immune-mediated neurological disorders. As such, myositis or Guillain-Barré-like syndromes are more frequently observed as chronic GVHD than MG (Maffini et al., 2017; Balaguer-Rosello et al., 2019).

## Miscellaneous and Unknown Mechanisms

A certain range of drugs have been associated with myasthenic side effects, but details regarding the underlying mechanisms are lacking in the literature. Hence, it can only be assumed based on pharmacological properties and the time course of neuromuscular manifestations, whether the effects relate to NMJ function or the immune system. Since the present article focuses on the underlying mechanisms of medication-associated myasthenia, such treatments with poorly characterized or unknown mechanisms will only be mentioned briefly, focusing on clinical aspects.

### Some Antibiotics

The aminopenicillin antibiotics ampicillin and amoxicillin, have anecdotally been associated with myasthenic weakness. Most recently, a series of six cases indicated symptom onset within few days after drug application, subsequently leading to clinical worsening, followed by symptom recovery after pyridostigmine and IVIg (Vacchiano et al., 2020). Moreover, ampicillin increased the degree of decrement in rabbits with experimental autoimmune myasthenia gravis but had no negative effects in normal rabbits (Argov et al., 1986).

Further, the macrolide antibiotics, which inhibit protein synthesis by binding to ribosomal structures, are broadly available for a wide range of infectious diseases. Occasional anecdotal reports have described a mild to moderate symptom aggravation by some macrolides (e.g., azithromycin, erythromycin) in myasthenic patients (May and Calvert, 1990; Cadisch et al., 1996). Albeit evidence is weak and no clear recommendations exist, patients with MG should be monitored closely under a treatment with these antibiotics.

### Corticosteroids

Corticosteroids require specific attention with regard to MG, since they are frequently used as a standard treatment. In experimental models, complex influences on the NMJ due to corticosteroids have been shown, including altered depolarization of nerve terminals, a reduction of ACh release, altered MEPPs, changes in choline transport and depletion of potassium (Wilson et al., 1974; Dengler et al., 1979; Kim et al., 1979). Yet, it is not far to seek that immunological mechanisms may also be involved in this phenomenon. Notably, one study found that an early clinical deterioration under corticosteroids was accompanied by a transient increase in lymphocyte response *in vitro*, stressing a role of cell-mediated immunological mechanisms (Abramsky et al., 1975).

Almost 50% of MG patients with high-dose steroid treatment experience an initial exacerbation of neuromuscular symptoms, of which 8.6% are severe, requiring mechanical ventilation or intubation (Pascuzzi et al., 1984). This potentially life-threatening complication can be avoided by gradually increasing the dosage, starting with 25 mg every other day (Seybold and Drachman, 1974). Apart from high initial dosages, thymoma, early-onset disease and upper limb weakness have been associated with steroid-induced worsening of myasthenia (Kanai et al., 2019).

### Hormonal Disturbances

Autoimmune thyroid disorders such as Grave’s disease and Hashimoto thyroiditis are more prevalent in individuals with MG (Song et al., 2019). A causal relation has been proposed, as symptoms were reported to fluctuate depending on thyroid hormone levels. However, systematic data corroborating this relationship are still lacking (Maclean and Wilson, 1954; Mallikarjuna et al., 2019).

It is also a frequently observed phenomenon that some female patients with autoimmune MG experience fluctuating weakness with the menstrual cycle and pregnancy, suggesting some modifying effects of female sex hormones. There is some evidence that a higher rate of remissions during menstruation may be due to reduced activity of acetylcholinesterase (Vijayan et al., 1977). While robust clinical data are missing to draw any definite conclusions, experimental data show conflicting results regarding the effect of sex hormones. First, one group used a rat model with experimental MG to analyze whether MG aggravation may be induced by changes in female sex hormones in rodents with and without ovariectomy. No electrophysiological and serological (AChR Abs) changes were observed after hormonal replacement, indicating no major influence on the susceptibility and severity of MG (Leker et al., 2000). By contrast, another work using an animal model showed that a temporary exposure to beta-17-estradiol enhances the production of anti-AChR Abs and significantly increases the severity of EAMG in mice, providing evidence that estrogen aggravates experimental MG (Delpy et al., 2005). Clinically, the notion that female sex hormones *per se* increase the susceptibility to autoimmune MG is supported by case descriptions (Brittain and Lange, 1995).

### Lithium

Lithium salts such as lithium carbonate, which are used as mood-stabilizing drugs, have been reported to either aggravate weakness in MG or unmask an autoimmune myasthenic syndrome (Neil et al., 1976; Lipton, 1987; Ronzière et al., 2000). The drugs have been shown to carry an inherent potential to foster T-lymphocytes activity, which is in favor of an immune-mediated mechanism as the main actor (Mizuno et al., 1982). However, lithium also appears to selectively reduce AChR synthesis and its insertion into the cell membrane (Pestronk and Drachman, 1987). It remains open to speculation, if these effects occur secondarily to an immunological mechanism.

### Iodinated Contrast Agents

Iodinated contrast agents (ICAs), which are commonly used for computed tomography (CT) scans, have for a long time been regarded as potential triggers of myasthenic symptoms. ICAs are subdivided into high- and low-osmolality compounds, and those with a high degree of osmolality (i.e., >1500 mOsm/L) are not in clinical use any more. Instead, low-osmolality agents (290–860 mOsm/L) are now routinely used due to their better tolerability (Pasternak and Williamson, 2012).

Several case reports and small case series as well as experimental data in rabbits suggested worsening of pre-existing MG related to the use of high-osmolality agents (Chagnac et al., 1985; Anzola et al., 1986; Bonmarchand et al., 1987; Eliashiv et al., 1990; Rocha and Bacheschi, 1994). Aside from anecdotal evidence, there has been one systematic trial in 136 patients retrospectively investigating acute events within 24 h after receiving high-osmolality agents, showing that 7 patients (5.1%) experienced an exacerbation of myasthenic weakness, whereas in the majority of cases, alternative explanations other than ICA were also conceivable (Frank et al., 1987).

With regard to the nowadays more commonly used low-osmolality ICAs, there are only a few publications to date. One study focusing on adverse reactions occurring just after ICA application reported a rate below 1%, hence suggesting no significant immediate risk due to neuromuscular blockade for MG patients (Mehrizi and Pascuzzi, 2014). By contrast, another retrospective work with an extended observation period found significantly more MG-related exacerbations within the first day after the contrast-enhanced scans, mainly respiratory manifestations, while there was no significant difference between the contrast and the non-contrast group thereafter (up to 45 days after the radiological investigation) (Somashekar et al., 2013). Most recently, one study retrospectively investigated acute and delayed aggravations of myasthenic symptoms in 72 patients with MG and found an increased rate (12.3%) of delayed exacerbations of MG after the application of low-osmolality ICAs, again stressing the high likelihood of alternative causes (unrelated to MG) for symptom exacerbation in the majority of cases (Rath et al., 2017).

Based upon the existing evidence, ICA administration should not be considered contraindicated in individuals with MG, as long as the patients are monitored regarding immediate and delayed adverse reactions. However, published results are somewhat contradictory, and large prospective studies are required for clarification.

## Conclusion

Our comprehensive review of the existing literature underscores that medication-related aggravations of myasthenic syndromes due to various molecular mechanisms represent a clinically relevant problem. The evidence for specific classes of drugs or treatments is often scanty and merely based on single case descriptions. First, one must be extremely cautious not to overinterpret anecdotal observations or suggestive (but unspecific) functional data, and risks and benefits need to be weighed carefully. Likewise, it is extremely important that appropriate treatments are not withheld because of anecdotal evidence only.

Apart from drug-related aggravations, our article also highlights a probably still underappreciated phenomenon, that is the induction of *de novo* myasthenia due to drugs with immune-modulatory properties. We specifically stress the emerging group of ICIs as a relatively novel etiology of drug-induced MG, which is particularly characterized by a rapidly progressive and potentially lethal clinical course.

Taken all together, it is obvious that more systematic data are required to estimate the associated risks of specific medical treatments more precisely. However, the thorough knowledge of cases already reported in the literature and the underlying molecular mechanisms may relevantly increase awareness of treating clinicians, so that patients can be monitored carefully, if potentially hazardous drugs have to be used.

## Author Contributions

MK drafted the manuscript. AG provided specific expertise for the chapter on ICIs. PW provided expertise regarding allogeneic HSCT. JR critically revised the article and provided specific input with regard to iodinated contrast agents. HC critically revised the manuscript and provided specific input regarding beta blocker-related mechanisms. IK designed the two figures with Biorender and critically revised the manuscript. FZ proposed and supervised the manuscript. All authors contributed to the article and approved the submitted version.

## Conflict of Interest

The authors declare that the research was conducted in the absence of any commercial or financial relationships that could be construed as a potential conflict of interest.

## References

[B1] Abramsky O. Aharonov A. Teitelbaum D. Fuchs S. (1975). Myasthenia gravis and acetylcholine receptor. Effect of steroids in clinical course and cellular immune response to acetylcholine receptor. *Arch. Neurol.* 32 684–687. 10.1001/archneur.1975.00490520054008 1180731

[B2] Adelman H. M. Winters P. R. Mahan C. S. Wallach P. M. (1995). D-penicillamine-induced myasthenia gravis: diagnosis obscured by coexisting chronic obstructive pulmonary disease. *Am. J. Med. Sci.* 309 191–193. 10.1097/00000441-199504000-00001 7900739

[B3] Albers J. W. Beals C. A. Levine S. P. (1981). Neuromuscular transmission in rheumatoid arthritis, with and without penicillamine treatment. *Neurology* 31 1562–1564. 10.1212/wnl.31.12.1562 6273769

[B4] Aldrich M. S. Kim Y. I. Sanders D. B. (1979). Effects of D-penicillamine on neuromuscular transmission in rats. *Muscle Nerve* 2 180–185. 10.1002/mus.880020305 228187

[B5] Alfrey A. C. Terman D. S. Brettschneider L. Simpson K. M. Ogden D. A. (1970). Hypermagnesemia after renal homotransplantation. *Ann. Intern. Med.* 73 367–371. 10.7326/0003-4819-73-3-367 4917178

[B6] Andonopoulos A. P. Terzis E. Tsibri E. Papasteriades C. A. Papapetropoulos T. (1994). D-penicillamine induced myasthenia gravis in rheumatoid arthritis: an unpredictable common occurrence? *Clin. Rheumatol.* 13 586–588. 10.1007/bf02242998 7697959

[B7] Anzola G. P. Capra R. Magoni M. Vignolo L. A. (1986). Myasthenic crisis during intravenous iodinated contrast medium injection. *Ital. J. Neurol. Sci.* 7 273. 10.1007/bf02230893 3721837

[B8] Argov Z. Brenner T. Abramsky O. (1986). Ampicillin may aggravate clinical and experimental myasthenia gravis. *Arch. Neurol.* 43 255–256. 10.1001/archneur.1986.00520030045010 3947272

[B9] Azevedo E. Ribeiro J. A. Polónia J. Pontes C. (1993). Probable exacerbation of myasthenia gravis by ofloxacin. *J. Neurol.* 240 508–508. 10.1007/bf00874123 8263560

[B10] Baik S. J. Kim T. H. Kim H. I. Rhie J. Y. (2016). Myasthenia crisis induced by pegylated-interferon in patient with chronic hepatitis c: a case report. *Medicine (Baltimore)* 95:e3782. 10.1097/MD.0000000000003782 27227948 PMC4902372

[B11] Balaguer-Rosello A. Bataller L. Piñana J. L. Montoro J. Lorenzo I. Villalba A. (2019). Noninfectious neurologic complications after allogeneic hematopoietic stem cell transplantation. *Biol. Blood Marrow Transplant.* 25 1818–1824. 10.1016/j.bbmt.2019.05.024 31132454

[B12] Batocchi A. P. Evoli A. Servidei S. Palmisani M. T. Apollo F. Tonali P. (1995). Myasthenia gravis during interferon alfa therapy. *Neurology* 45 382–383. 10.1212/wnl.45.2.382 7854543

[B13] Bever C. T. Chang H. W. Penn A. S. Jaffe I. A. Bock E. (1982). Penicillamine-induced myasthenia gravis: effects of penicillamine on acetylcholine receptor. *Neurology* 32 1077–1082. 10.1212/wnl.32.10.1077 6889694

[B14] Blake G. Murphy S. (1997). Onset of myasthenia gravis in a patient with multiple sclerosis during interferon-1b treatment. *Neurology* 49 1747–1748. 10.1212/wnl.49.6.1747-a 9409387

[B15] Blichfeldt-Lauridsen L. Hansen B. D. (2012). Anesthesia and myasthenia gravis. *Acta Anaesthesiol. Scand.* 56 17–22. 10.1111/j.1399-6576.2011.02558.x 22091897

[B16] Bolger G. B. Sullivan K. M. Spence A. M. Appelbaum F. R. Johnston R. Sanders J. E. (1986). Myasthenia gravis after allogeneic bone marrow transplantation: relationship to chronic graft-versus-host disease. *Neurology* 36 1087–1091. 10.1212/wnl.36.8.1087 3526178

[B17] Bonmarchand G. Weiss P. Clavier E. Lerebours-Pigeonniere G. Massari P. Leroy J. (1987). Myasthenic crisis following the injection of an iodinated contrast medium. *Intensive Care Med.* 13:365. 3655107

[B18] Booij L. H. Miller R. D. Crul J. F. (1978). Neostigmine and 4-aminopyridine antagonism of lincomycin-pancuronium neuromuscular blockade in man. *Anesth. Analg.* 57 316–321. 10.1213/00000539-197805000-00007 566050

[B19] Borgia G. Reynaud L. Gentile I. Cerini R. Ciampi R. Russo M. D. (2001). Myasthenia gravis during low-dose IFN-alpha therapy for chronic hepatitis C. *J. Interferon Cytokine Res.* 21 469–470. 10.1089/10799900152434321 11506739

[B20] Brittain J. Lange L. S. (1995). Myasthenia gravis and levonorgestrel implant. *Lancet* 346:1556. 10.1016/s0140-6736(95)92084-6 7491061

[B21] Brumlik J. Jacobs R. S. (1974). Myasthenia gravis associated with diphenylhydantoin therapy for epilepsy. *Can. J. Neurol. Sci.* 1 127–129. 10.1017/s0317167100019697 4215556

[B22] Bruna J. Argyriou A. A. Anastopoulou G. G. Alemany M. Nadal E. Kalofonou F. (2020). Incidence and characteristics of neurotoxicity in immune checkpoint inhibitors with focus on neuromuscular events: experience beyond the clinical trials. *J. Peripher. Nerv. Syst.* 25 171–177. 10.1111/jns.12371 32166812

[B23] Cadisch R. Streit E. Hartmann K. (1996). [Exacerbation of pseudoparalytic myasthenia gravis following azithromycin (Zithromax)]. *Schweiz Med. Wochenschr* 126 308–310. 8701248

[B24] Caputy A. J. Kim Y. I. Sanders D. B. (1981). The neuromuscular blocking effects of therapeutic concentrations of various antibiotics on normal rat skeletal muscle: a quantitative comparison. *J. Pharmacol. Exp. Ther.* 217 369–378. 6112258

[B25] Cartwright M. S. Jeffery D. R. Nuss G. R. Donofrio P. D. (2004). Statin-associated exacerbation of myasthenia gravis. *Neurology* 63 2188–2188. 10.1212/01.wnl.0000145708.03876.c3 15596782

[B26] Cata J. P. Lasala J. D. Williams W. Mena G. E. (2019). Myasthenia gravis and thymoma surgery: a clinical update for the cardiothoracic anesthesiologist. *J. Cardiothorac. Vasc. Anesth.* 33 2537–2545. 10.1053/j.jvca.2018.07.036 30219643

[B27] Cetin H. Vincent A. (2018). Pathogenic mechanisms and clinical correlations in autoimmune myasthenic syndromes. *Semin. Neurol.* 38 344–354. 10.1055/s-0038-1660500 30011414

[B28] Chagnac Y. Hadani M. Goldhammer Y. (1985). Myasthenic crisis after intravenous administration of iodinated contrast agent. *Neurology* 35 1219–1220. 10.1212/wnl.35.8.1219 4022360

[B29] Choi J. Lee S. Y. (2020). Clinical characteristics and treatment of immune-related adverse events of immune checkpoint inhibitors. *Immune Netw.* 20:e9. 10.4110/in.2020.20.e9 32158597 PMC7049586

[B30] Clausen L. Cossins J. Beeson D. (2018). Beta-2 adrenergic receptor agonists enhance AChR clustering in C2C12 myotubes: implications for therapy of myasthenic disorders. *J. Neuromuscul. Dis.* 5 231–240. 10.3233/JND-170293 29865088 PMC6004912

[B31] Cohen B. A. London R. S. Goldstein P. J. (1976). Myasthenia gravis and preeclampsia. *Obstet. Gynecol.* 48 35S–37S. 940634

[B32] Confavreux C. Charles N. Aimard G. (1990). Fulminant myasthenia gravis soon after initiation of acebutolol therapy. *Eur. Neurol.* 30 279–281. 10.1159/000117380 2269319

[B33] Congeni J. P. Kirkpatrick R. B. (2013). Pegylated interferon induced myasthenia crisis–a case report. *J. Clin. Neuromuscul. Dis.* 14 123–125. 10.1097/CND.0b013e318285257f 23492465

[B34] Conlon K. C. Urba W. J. Smith J. W. Steis R. G. Longo D. L. Clark J. W. (1990). Exacerbation of symptoms of autoimmune disease in patients receiving alpha-interferon therapy. *Cancer* 65 2237–2242. 10.1002/1097-0142(19900515)65:10<2237::aid-cncr2820651013<3.0.co;2-5 2346907

[B35] Coppeto J. R. (1984). Timolol-associated myasthenia gravis. *Am. J. Ophthalmol.* 98 244–245. 10.1016/0002-9394(87)90366-7 6476051

[B36] Dashtipour K. Pedouim F. (2016). Botulinum toxin: preparations for clinical use, immunogenicity, side effects, and safety profile. *Semin. Neurol.* 36 29–33. 10.1055/s-0035-1571213 26866493

[B37] De Sarro A. De Sarro G. (2001). Adverse reactions to fluoroquinolones. an overview on mechanistic aspects. *Curr. Med. Chem.* 8 371–384. 10.2174/0929867013373435 11172695

[B38] del Castillo J. Engbaek L. (1954). The nature of the neuromuscular block produced by magnesium. *J. Physiol. (Lond.)* 124 370–384. 10.1113/jphysiol.1954.sp005114 13175138 PMC1366273

[B39] Delpy L. Douin-Echinard V. Garidou L. Bruand C. Saoudi A. Guéry J.-C. (2005). Estrogen enhances susceptibility to experimental autoimmune myasthenia gravis by promoting type 1-polarized immune responses. *J. Immunol.* 175 5050–5057. 10.4049/jimmunol.175.8.5050 16210608

[B40] Dengler R. Rüdel R. Warelas J. Birnberger K. L. (1979). Corticosteroids and neuromuscular transmission: electrophysiological investigation of the effects of prednisolone on normal and anticholinesterase-treated neuromuscular junction. *Pflugers Arch.* 380 145–151. 10.1007/bf00582150 225724

[B41] Dionisiotis J. Zoukos Y. Thomaides T. (2004). Development of myasthenia gravis in two patients with multiple sclerosis following interferon beta treatment. *J. Neurol. Neurosurg. Psychiatry* 75 1079–1079. 10.1136/jnnp.2003.028233 15201386 PMC1739135

[B42] Dolly J. O. Aoki K. R. (2006). The structure and mode of action of different botulinum toxins. *Eur. J. Neurol.* 13(Suppl. 4) 1–9. 10.1111/j.1468-1331.2006.01648.x 17112344

[B43] Drosos A. A. Christou L. Galanopoulou V. Tzioufas A. G. Tsiakou E. K. (1993). D-penicillamine induced myasthenia gravis: clinical, serological and genetic findings. *Clin. Exp. Rheumatol.* 11 387–391. 8403583

[B44] Durant N. N. Lambert J. J. (1981). The action of polymyxin B at the frog neuromuscular junction. *Br. J. Pharmacol.* 72 41–47. 10.1111/j.1476-5381.1981.tb09102.x 6112033 PMC2071540

[B45] Eliashiv S. Wirguin I. Brenner T. Argov Z. (1990). Aggravation of human and experimental myasthenia gravis by contrast media. *Neurology* 40 1623–1625. 10.1212/wnl.40.10.1623 2215958

[B46] Elmqvist D. Hofmann W. W. Kugelberg J. Quastel D. M. (1964). An electrophysiological investigation of neuromuscular transmission in myasthenia gravis. *J. Physiol.* 174 417–434. 10.1113/jphysiol.1964.sp007495 14232401 PMC1368938

[B47] Erbguth F. Claus D. Engelhardt A. Dressler D. (1993). Systemic effect of local botulinum toxin injections unmasks subclinical Lambert-Eaton myasthenic syndrome. *J. Neurol. Neurosurg. Psychiatry* 56 1235–1236. 10.1136/jnnp.56.11.1235 8229041 PMC489831

[B48] Evans M. Rees A. (2002). Effects of HMG-CoA reductase inhibitors on skeletal muscle: are all statins the same? *Drug Saf.* 25 649–663. 10.2165/00002018-200225090-00004 12137559

[B49] Fife B. T. Bluestone J. A. (2008). Control of peripheral T-cell tolerance and autoimmunity via the CTLA-4 and PD-1 pathways. *Immunol. Rev.* 224 166–182. 10.1111/j.1600-065X.2008.00662.x 18759926

[B50] Fishman R. A. (1965). Neurological aspects of magnesium metabolism. *Arch. Neurol.* 12 562–569. 10.1001/archneur.1965.00460300010002 14295956

[B51] Frank J. H. Cooper G. W. Black W. C. Phillips L. H. (1987). Iodinated contrast agents in myasthenia gravis. *Neurology* 37 1400–1402. 10.1212/wnl.37.8.1400 3614668

[B52] Gage P. W. Hamill O. P. (1976). Effects of several inhalation anaesthetics on the kinetics of postsynaptic conductance changes in mouse diaphragm. *Br. J. Pharmacol.* 57 263–272. 10.1111/j.1476-5381.1976.tb07476.x 938796 PMC1667113

[B53] Gale J. Danesh-Meyer H. V. (2014). Statins can induce myasthenia gravis. *J. Clin. Neurosci.* 21 195–197. 10.1016/j.jocn.2013.11.009 24433954

[B54] Garner C. G. Straube A. Witt T. N. Gasser T. Oertel W. H. (1993). Time course of distant effects of local injections of botulinum toxin. *Mov. Disord.* 8 33–37. 10.1002/mds.870080106 8380486

[B55] Godley P. J. Morton T. A. Karboski J. A. Tami J. A. (1990). Procainamide-induced myasthenic crisis. *Ther. Drug Monit.* 12 411–414. 10.1097/00007691-199007000-00019 2396315

[B56] Gu D. Wogensen L. Calcutt N. A. Xia C. Zhu S. Merlie J. P. (1995). Myasthenia gravis-like syndrome induced by expression of interferon gamma in the neuromuscular junction. *J. Exp. Med.* 181 547–557. 10.1084/jem.181.2.547 7836911 PMC2191877

[B57] Guidon A. C. (2019). Lambert-eaton myasthenic syndrome, botulism, and immune checkpoint inhibitor-related myasthenia gravis. *Continuum (Minneap Minn)* 25 1785–1806. 10.1212/CON.0000000000000807 31794471

[B58] Gummi R. R. Kukulka N. A. Deroche C. B. Govindarajan R. (2019). Factors associated with acute exacerbations of myasthenia gravis. *Muscle Nerve* 60 693–699. 10.1002/mus.26689 31469909

[B59] Gunduz A. Turedi S. Kalkan A. Nuhoglu I. (2006). Levofloxacin induced myasthenia crisis. *Emerg. Med. J.* 23 662–662. 10.1136/emj.2006.038091 16858118 PMC2564188

[B60] Gutmann L. Takamori M. (1973). Effect of Mg++ on neuromuscular transmission in the Eaton-Lambert syndrome. *Neurology* 23 977–980. 10.1212/wnl.23.9.977 4353553

[B61] Harry J. D. Linden R. J. Snow H. M. (1974). The effects of three beta-adrenoceptor blocking drugs on isolated preparations of skeletal and cardiac muscle. *Br. J. Pharmacol.* 52 275–281. 10.1111/j.1476-5381.1974.tb09710.x 4155991 PMC1776866

[B62] Haugh A. M. Probasco J. C. Johnson D. B. (2020). Neurologic complications of immune checkpoint inhibitors. *Expert Opin. Drug Saf.* 18 1–10. 10.1080/14740338.2020.1738382 32126176 PMC7192781

[B63] Huda S. Cao M. De Rosa A. Woodhall M. Rodríguez Cruz P. M. Cossins J. (2020). SHP2 inhibitor protects AChRs from effects of myasthenia gravis MuSK antibody. *Neurol. Neuroimmunol. Neuroinflamm.* 7:e645. 10.1212/NXI.0000000000000645 31831571 PMC6935836

[B64] Jamal B. T. Herb K. (2009). Perioperative management of patients with myasthenia gravis: prevention, recognition, and treatment. *Oral Surg. Oral Med. Oral Pathol. Oral Radiol. Endod.* 107 612–615. 10.1016/j.tripleo.2009.01.015 19426917

[B65] Jennett A. M. Bali D. Jasti P. Shah B. Browning L. A. (2006). Telithromycin and myasthenic crisis. *Clin. Infect. Dis.* 43 1621–1622. 10.1086/509646 17109301

[B66] Johansen A. Christensen S. J. Scheie D. Højgaard J. L. S. Kondziella D. (2019). Neuromuscular adverse events associated with anti-PD-1 monoclonal antibodies: systematic review. *Neurology* 92 663–674. 10.1212/WNL.0000000000007235 30850443

[B67] Johnson D. B. Manouchehri A. Haugh A. M. Quach H. T. Balko J. M. Lebrun-Vignes B. (2019). Neurologic toxicity associated with immune checkpoint inhibitors: a pharmacovigilance study. *J. Immunother. Cancer* 7:134. 10.1186/s40425-019-0617-x 31118078 PMC6530194

[B68] Johnson D. B. Saranga-Perry V. Lavin P. J. M. Burnette W. B. Clark S. W. Uskavitch D. R. (2015). Myasthenia gravis induced by ipilimumab in patients with metastatic melanoma. *J. Clin. Oncol.* 33 e122–e124. 10.1200/JCO.2013.51.1683 24778401 PMC4979104

[B69] Jonkers I. Swerup C. Pirskanen R. Bjelak S. Matell G. (1996). Acute effects of intravenous injection of beta-adrenoreceptor- and calcium channel at antagonists and agonists in myasthenia gravis. *Muscle Nerve* 19 959–965. 10.1002/(SICI)1097-4598(199608)19:8<959::AID-MUS4<3.0.CO;2-7 8756161

[B70] Kaeser H. E. (1984). Drug-induced myasthenic syndromes. *Acta Neurol. Scand. Suppl.* 100 39–47. 6148832

[B71] Kanai T. Uzawa A. Kawaguchi N. Oda F. Ozawa Y. Himuro K. (2019). Predictive score for oral corticosteroid-induced initial worsening of seropositive generalized myasthenia gravis. *J. Neurol. Sci.* 396 8–11. 10.1016/j.jns.2018.10.018 30391823

[B72] Khalid R. Ibad A. Thompson P. D. (2016). Statins and myasthenia gravis. *Muscle Nerve* 54 509–509. 10.1002/mus.25155 27105400

[B73] Khella S. L. Kozart D. (1997). Unmasking and exacerbation of myasthenia gravis by ophthalmic solutions: betoxolol, tobramycin, and dexamethasone. A case report. *Muscle Nerve* 20 631–631. 10.1002/mus.880200501 9140379

[B74] Kim Y. I. Goldner M. M. Sanders D. B. (1979). Short-term effects of prednisolone on neuromuscular transmission in normal rats and those with experimental autoimmune myasthenia gravis. *J. Neurol. Sci.* 41 223–234. 10.1016/0022-510x(79)90041-8 220391

[B75] Kimbara S. Fujiwara Y. Iwama S. Ohashi K. Kuchiba A. Arima H. (2018). Association of antithyroglobulin antibodies with the development of thyroid dysfunction induced by nivolumab. *Cancer Sci.* 109 3583–3590. 10.1111/cas.13800 30230649 PMC6215874

[B76] Komal Kumar R. N. Patil S. A. Taly A. B. Nirmala M. Sinha S. Arunodaya G. R. (2004). Effect of D-penicillamine on neuromuscular junction in patients with Wilson disease. *Neurology* 63 935–936. 10.1212/01.wnl.0000137021.90567.37 15365158

[B77] Koneczny I. Herbst R. (2019). Myasthenia gravis: pathogenic effects of autoantibodies on neuromuscular architecture. *Cells* 8:671. 10.3390/cells8070671 31269763 PMC6678492

[B78] Krendel D. A. (1990). Hypermagnesemia and neuromuscular transmission. *Semin. Neurol.* 10 42–45. 10.1055/s-2008-1041252 2161126

[B79] Krendel D. A. Hopkins L. C. (1986). Adverse effect of verapamil in a patient with the Lambert-Eaton syndrome. *Muscle Nerve* 9 519–522. 10.1002/mus.880090607 3016531

[B80] Kuncl R. W. Pestronk A. Drachman D. B. Rechthand E. (1986). The pathophysiology of penicillamine-induced myasthenia gravis. *Ann. Neurol.* 20 740–744. 10.1002/ana.410200617 3813503

[B81] Lee D. C. Kim Y. I. Liu H. H. Johns T. R. (1983). Presynaptic and postsynaptic actions of procainamide on neuromuscular transmission. *Muscle Nerve* 6 442–447. 10.1002/mus.880060608 6312309

[B82] Lee M. Beeson D. Palace J. (2018). Therapeutic strategies for congenital myasthenic syndromes. *Ann. N.Y. Acad. Sci.* 1412 129–136. 10.1111/nyas.13538 29381222

[B83] Lefvert A. K. Björkholm M. (1987). Antibodies against the acetylcholine receptor in hematologic disorders: implications for the development of myasthenia gravis after bone marrow grafting. *N. Engl. J. Med.* 317 170–171. 10.1056/NEJM198707163170315 3299087

[B84] Leker R. R. Karni A. Brenner T. Weidenfeld J. Abramsky O. (2000). Effects of sex hormones on experimental autoimmune myasthenia gravis. *Eur. J. Neurol.* 7 203–206. 10.1046/j.1468-1331.2000.00042.x 10809942

[B85] Leys D. Pasquier F. Vermersch P. Gosset D. Michiels H. Kassiotis P. (1987). Possible revelation of latent myasthenia gravis by labetalol chlorhydrate. *Acta Clin. Belg.* 42 475–476. 10.1080/22953337.1987.11719269 3434123

[B86] Lipsitz P. J. (1971). The clinical and biochemical effects of excess magnesium in the newborn. *Pediatrics* 47 501–509. 5547870

[B87] Lipton I. D. (1987). Myasthenia gravis unmasked by lithium carbonate. *J. Clin. Psychopharmacol.* 7:57. 10.1097/00004714-198702000-00030 3102565

[B88] Liu C.-N. Somps C. J. (2010). Telithromycin blocks neuromuscular transmission and inhibits nAChR currents in vitro. *Toxicol. Lett.* 194 66–69. 10.1016/j.toxlet.2010.02.005 20153815

[B89] Maclean B. Wilson J. A. (1954). See-saw relationship between hyperthyroidism and myasthenia gravis. *Lancet* 266 950–953. 10.1016/s0140-6736(54)91566-4 13164301

[B90] Maffini E. Festuccia M. Brunello L. Boccadoro M. Giaccone L. Bruno B. (2017). Neurologic complications after allogeneic hematopoietic stem cell transplantation. *Biol. Blood Marrow Transplant.* 23 388–397. 10.1016/j.bbmt.2016.12.632 28039081

[B91] Maimone M. M. Merlie J. P. (1993). Interaction of the 43 kd postsynaptic protein with all subunits of the muscle nicotinic acetylcholine receptor. *Neuron* 11 53–66. 10.1016/0896-6273(93)90270-2 8338668

[B92] Mallikarjuna S. K. Velayutham S. S. Sowmini P. R. Jeyaraj M. K. Arunan S. (2019). See-saw relationship and its reversal after immunotherapy in a case of Graves’ disease with coexisting myasthenia gravis. *J. Neurosci. Rural Pract.* 10 136–138. 10.4103/jnrp.jnrp_150_18 30765989 PMC6337967

[B93] May E. F. Calvert P. C. (1990). Aggravation of myasthenia gravis by erythromycin. *Ann. Neurol.* 28 577–579. 10.1002/ana.410280417 2252369

[B94] Mehra M. R. Desai S. S. Ruschitzka F. Patel A. N. (2020). Hydroxychloroquine or chloroquine with or without a macrolide for treatment of COVID-19: a multinational registry analysis. *Lancet* 395:1820 10.1016/S0140-6736(20)31180-6 PMC7274621 32511943

[B95] Mehrizi M. Pascuzzi R. M. (2014). Complications of radiologic contrast in patients with myasthenia gravis. *Muscle Nerve* 50 443–444. 10.1002/mus.24254 24677227

[B96] Michot J. M. Bigenwald C. Champiat S. Collins M. Carbonnel F. Postel-Vinay S. (2016). Immune-related adverse events with immune checkpoint blockade: a comprehensive review. *Eur. J. Cancer* 54 139–148. 10.1016/j.ejca.2015.11.016 26765102

[B97] Milani M. Ostlie N. Wang W. Conti-Fine B. M. (2003). T cells and cytokines in the pathogenesis of acquired myasthenia gravis. *Ann. N.Y. Acad. Sci.* 998 284–307. 10.1196/annals.1254.032 14592887

[B98] Miller C. D. Oleshansky M. A. Gibson K. F. Cantilena L. R. (1993). Procainamide-induced myasthenia-like weakness and dysphagia. *Ther. Drug Monit.* 15 251–254. 10.1097/00007691-199306000-00013 7687390

[B99] Mitsune A. Yanagisawa S. Fukuhara T. Miyauchi E. Morita M. Ono M. (2018). Relapsed myasthenia gravis after nivolumab treatment. *Intern. Med.* 57 1893–1897. 10.2169/internalmedicine.9153-17 29434145 PMC6064691

[B100] Mizuno Y. Dosch H. M. Gelfand E. W. (1982). Carbamycholine modulation of E-rosette formation: identification of nicotinic acetylcholine receptors on a subpopulation of human T lymphocytes. *J. Clin. Immunol.* 2 303–308. 10.1007/bf00915071 6982902

[B101] Montes V. Sousa S. Pita F. Guerreiro R. Carmona C. (2018). Myasthenia gravis induced by Ipilimumab in a patient with metastatic melanoma. *Front. Neurol.* 9:150. 10.3389/fneur.2018.00150 29666602 PMC5891586

[B102] Moore B. Safani M. Keesey J. (1988). Possible exacerbation of myasthenia gravis by ciprofloxacin. *Lancet* 1:882 10.1016/s0140-6736(88)91627-3 2895386

[B103] Mumford C. J. Ginsberg L. (1990). Ciprofloxacin and myasthenia gravis. *BMJ* 301 818–818. 10.1136/bmj.301.6755.818-a 2224281 PMC1663942

[B104] Neil J. F. Himmelhoch J. M. Licata S. M. (1976). Emergence of myasthenia gravis during treatment with lithium carbonate. *Arch. Gen. Psychiatry* 33 1090–1092. 10.1001/archpsyc.1976.01770090080007 962492

[B105] Nieman R. B. Sharma K. Edelberg H. Caffe S. E. (2003). Telithromycin and myasthenia gravis. *Clin. Infect. Dis.* 37 1579–1579. 10.1086/379617 14614683

[B106] Nilsson E. Meretoja O. A. (1990). Vecuronium dose-response and maintenance requirements in patients with myasthenia gravis. *Anesthesiology* 73 28–32. 10.1097/00000542-199007000-00005 1972873

[B107] Nitahara K. Sugi Y. Higa K. Shono S. Hamada T. (2007). Neuromuscular effects of sevoflurane in myasthenia gravis patients. *Br. J. Anaesth.* 98 337–341. 10.1093/bja/ael368 17251207

[B108] Norris F. H. Colella J. Mcfarlin D. (1964). Effect of diphenylhydantoin on neuromuscular synapse. *Neurology* 14 869–876. 10.1212/wnl.14.9.869 14215605

[B109] Oh S. J. Dhall R. Young A. Morgan M. B. Lu L. Claussen G. C. (2008). Statins may aggravate myasthenia gravis. *Muscle Nerve* 38 1101–1107. 10.1002/mus.21074 18720508 PMC2670554

[B110] Oh S. J. Elmore R. S. Sarala P. K. Kuba T. (1986). Procainamide-induced myasthenia-like syndrome. *Muscle Nerve* 9 670–672. 3762585

[B111] Parmar B. Francis P. J. Ragge N. K. (2002). Statins, fibrates, and ocular myasthenia. *Lancet* 360:717. 10.1016/S0140-6736(02)09846-X 12241896

[B112] Pascuzzi R. M. Coslett H. B. Johns T. R. (1984). Long-term corticosteroid treatment of myasthenia gravis: report of 116 patients. *Ann. Neurol.* 15 291–298. 10.1002/ana.410150316 6721451

[B113] Pasternak J. J. Williamson E. E. (2012). Clinical pharmacology, uses, and adverse reactions of iodinated contrast agents: a primer for the non-radiologist. *Mayo Clin. Proc.* 87 390–402. 10.1016/j.mayocp.2012.01.012 22469351 PMC3538464

[B114] Penn A. S. Low B. W. Jaffe I. A. Luo L. Jacques J. J. (1998). Drug-induced autoimmune myasthenia gravis. *Ann. N.Y. Acad. Sci.* 841 433–449. 10.1111/j.1749-6632.1998.tb10961.x 9668273

[B115] Perrot X. Bernard N. Vial C. Antoine J. C. Laurent H. Vial T. (2006). Myasthenia gravis exacerbation or unmasking associated with telithromycin treatment. *Neurology* 67 2256–2258. 10.1212/01.wnl.0000247741.72466.8c 17065592

[B116] Pestronk A. Drachman D. B. (1987). Mechanism of action of lithium on acetylcholine receptor metabolism in skeletal muscle. *Brain Res.* 412 302–310. 10.1016/0006-8993(87)91137-1 3038266

[B117] Peyer A.-K. Abicht A. Heinimann K. Sinnreich M. Fischer D. (2013). Quinine sulfate as a therapeutic option in a patient with slow channel congenital myasthenic syndrome. *Neuromuscul. Disord.* 23 571–574. 10.1016/j.nmd.2013.04.001 23688972

[B118] Pittinger C. Adamson R. (1972). Antibiotic blockade of neuromuscular function. *Annu. Rev. Pharmacol.* 12 169–184. 10.1146/annurev.pa.12.040172.001125 4261048

[B119] Postow M. A. Callahan M. K. Wolchok J. D. (2015). Immune checkpoint blockade in cancer therapy. *J. Clin. Oncol.* 33 1974–1982. 10.1200/JCO.2014.59.4358 25605845 PMC4980573

[B120] Postow M. A. Sidlow R. Hellmann M. D. (2018). Immune-related adverse events associated with immune checkpoint blockade. *N. Engl. J. Med.* 378 158–168. 10.1056/NEJMra1703481 29320654

[B121] Pritchard J. A. (1979). The use of magnesium sulfate in preeclampsia-eclampsia. *J. Reprod. Med.* 23 107–114. 490496

[B122] Protti D. A. Reisin R. Mackinley T. A. Uchitel O. D. (1996). Calcium channel blockers and transmitter release at the normal human neuromuscular junction. *Neurology* 46 1391–1396. 10.1212/wnl.46.5.1391 8628488

[B123] Purvin V. Kawasaki A. Smith K. H. Kesler A. (2006). Statin-associated myasthenia gravis: report of 4 cases and review of the literature. *Medicine (Baltimore)* 85 82–85. 10.1097/01.md.0000209337.59874.aa 16609346

[B124] Rath J. Mauritz M. Zulehner G. Hilger E. Cetin H. Kasprian G. (2017). Iodinated contrast agents in patients with myasthenia gravis: a retrospective cohort study. *J. Neurol.* 264 1209–1217. 10.1007/s00415-017-8518-8 28550477 PMC5486553

[B125] Ribera A. B. Nastuk W. L. (1989). The actions of verapamil at the neuromuscular junction. *Comp. Biochem. Physiol. C Comp. Pharmacol. Toxicol.* 93 137–141. 10.1016/0742-8413(89)90023-6 2567223

[B126] Robberecht W. Bednarik J. Bourgeois P. van Hees J. Carton H. (1989). Myasthenic syndrome caused by direct effect of chloroquine on neuromuscular junction. *Arch. Neurol.* 46 464–468. 10.1001/archneur.1989.00520400124033 2650665

[B127] Rocha M. S. Bacheschi L. A. (1994). Exacerbation of myasthenia gravis by contrast media. *AJR Am. J. Roentgenol.* 162 997. 10.2214/ajr.162.4.8141034 8141034

[B128] Ronzière T. Auzou P. Ozsancak C. Magnier P. Sénant J. Hannequin D. (2000). [Myasthenic syndrome induced by lithium]. *Presse Med.* 29 1043–1044. 10874911

[B129] Roquer J. Cano A. Seoane J. L. Pou Serradell A. (1996). Myasthenia gravis and ciprofloxacin. *Acta Neurol. Scand.* 94 419–420. 10.1111/j.1600-0404.1996.tb00055.x 9017031

[B130] Rubbo J. T. Gergis S. D. Sokoll M. D. (1977). Comparative neuromuscular effects of lincomycin and clindamycin. *Anesth. Analg.* 56 329–332. 10.1213/00000539-197705000-00001 194504

[B131] Safa H. Johnson D. H. Trinh V. A. Rodgers T. E. Lin H. Suarez-Almazor M. E. (2019). Immune checkpoint inhibitor related myasthenia gravis: single center experience and systematic review of the literature. *J. Immunother. Cancer* 7:319. 10.1186/s40425-019-0774-y 31753014 PMC6868691

[B132] Saleem A. (2016). Unmasking of myasthenia gravis during pegylated Alfa 2 a interferon and ribavirin therapy for chronic hepatitis C. *J. Pak. Med. Assoc.* 66 618–620. 27183950

[B133] Sato K. Mano T. Iwata A. Toda T. (2019). Neurological and related adverse events in immune checkpoint inhibitors: a pharmacovigilance study from the Japanese adverse drug event report database. *J. Neurooncol.* 145 1–9. 10.1007/s11060-019-03273-1 31452071

[B134] Schaller S. J. Lewald H. (2016). Clinical pharmacology and efficacy of sugammadex in the reversal of neuromuscular blockade. *Expert Opin. Drug Metab. Toxicol.* 12 1097–1108. 10.1080/17425255.2016.1215426 27463265

[B135] Seybold M. E. Drachman D. B. (1974). Gradually increasing doses of prednisone in myasthenia gravis. Reducing the hazards of treatment. *N. Engl. J. Med.* 290 81–84. 10.1056/NEJM197401102900204 4808454

[B136] Shi J. Niu J. Shen D. Liu M. Tan Y. Li Y. (2020). Clinical diagnosis and treatment recommendations for immune checkpoint inhibitor-related adverse reactions in the nervous system. *Thorac. Cancer* 11 481–487. 10.1111/1759-7714.13266 31823509 PMC6996972

[B137] Sieb J. P. Milone M. Engel A. G. (1996). Effects of the quinoline derivatives quinine, quinidine, and chloroquine on neuromuscular transmission. *Brain Res.* 712 179–189. 10.1016/0006-8993(95)01349-0 8814892

[B138] Singh P. Idowu O. Malik I. Nates J. L. (2015). Acute respiratory failure induced by magnesium replacement in a 62-year-old woman with myasthenia gravis. *Tex Heart Inst. J.* 42 495–497. 10.14503/THIJ-14-4584 26504451 PMC4591897

[B139] Singh Y. N. Harvey A. L. Marshall I. G. (1978a). Antibiotic-induced paralysis of the mouse phrenic nerve-hemidiaphragm preparation, and reversibility by calcium and by neostigmine. *Anesthesiology* 48 418–424. 10.1097/00000542-197806000-00008 208426

[B140] Singh Y. N. Marshall I. G. Harvey A. L. (1978b). Reversal of antibiotic-induced muscle paralysis by 3,4-diaminopyridine. *J. Pharm. Pharmacol.* 30 249–250. 10.1111/j.2042-7158.1978.tb13216.x 24716

[B141] Slater C. R. (2008). Reliability of neuromuscular transmission and how it is maintained. *Handb. Clin. Neurol.* 91 27–101. 10.1016/S0072-9752(07)01502-3 18631840

[B142] Somashekar D. K. Davenport M. S. Cohan R. H. Dillman J. R. Ellis J. H. (2013). Effect of intravenous low-osmolality iodinated contrast media on patients with myasthenia gravis. *Radiology* 267 727–734. 10.1148/radiol.12121508 23360741

[B143] Song R.-H. Yao Q.-M. Wang B. Li Q. Jia X. Zhang J.-A. (2019). Thyroid disorders in patients with myasthenia gravis: a systematic review and meta-analysis. *Autoimmun. Rev.* 18:102368. 10.1016/j.autrev.2019.102368 31404702

[B144] Swash M. Ingram D. A. (1992). Adverse effect of verapamil in myasthenia gravis. *Muscle Nerve* 15 396–398. 10.1002/mus.880150321 1557089

[B145] Swift T. R. (1979). Weakness from magnesium-containing cathartics: electrophysiologic studies. *Muscle Nerve* 2 295–298. 10.1002/mus.880020409 492206

[B146] Timmermans G. Depierreux F. Wang F. Hansen I. Maquet P. (2019). Cosmetic injection of botulinum toxin unmasking subclinical myasthenia gravis: a case report and literature review. *Case Rep. Neurol.* 11 244–251. 10.1159/000502350 31572161 PMC6751432

[B147] Titulaer M. J. Lang B. Verschuuren J. J. (2011). Lambert-Eaton myasthenic syndrome: from clinical characteristics to therapeutic strategies. *Lancet Neurol.* 10 1098–1107. 10.1016/S1474-4422(11)70245-9 22094130

[B148] Touret F. de Lamballerie X. (2020). Of chloroquine and COVID-19. *Antiviral Res.* 177:104762. 10.1016/j.antiviral.2020.104762 32147496 PMC7132364

[B149] Tsutsumi Y. Kamiishi T. Kikuchi R. Ito S. Matsuoka S. Teshima T. (2019). Myasthenia gravis after allogeneic bone marrow transplantation: a case report and literature review. *Hematol. Oncol. Stem Cell Ther.* 12 110–114. 10.1016/j.hemonc.2017.04.001 28549768

[B150] Vacchiano V. Solli P. Bartolomei I. Lai G. Liguori R. Salvi F. (2020). Exacerbation of myasthenia gravis after amoxicillin therapy: a case series. *Neurol. Sci.* 41 2255–2257. 10.1007/s10072-020-04387-5 32296986

[B151] Vaklavas C. Chatzizisis Y. S. Ziakas A. Zamboulis C. Giannoglou G. D. (2009). Molecular basis of statin-associated myopathy. *Atherosclerosis* 202 18–28. 10.1016/j.atherosclerosis.2008.05.021 18585718

[B152] Vanhaesebrouck A. E. Beeson D. (2019). The congenital myasthenic syndromes: expanding genetic and phenotypic spectrums and refining treatment strategies. *Curr. Opin. Neurol.* 32 696–703. 10.1097/WCO.0000000000000736 31361628 PMC6735524

[B153] Varan O. Kucuk H. Tufan A. (2015). Myasthenia gravis due to hydroxychloroquine. *Reumatismo* 67:849. 10.4081/reumatismo.2015.849 26876193

[B154] Verkijk A. (1985). Worsening of myasthenia gravis with timolol maleate eyedrops. *Ann. Neurol.* 17 211–212. 10.1002/ana.410170222 3977305

[B155] Vijayan N. Vijayan V. K. Dreyfus P. M. (1977). Acetylcholinesterase activity and menstrual remissions in myasthenia gravis. *J. Neurol. Neurosurg. Psychiatry* 40 1060–1065. 10.1136/jnnp.40.11.1060 599353 PMC492903

[B156] Vincent A. Newsom-Davis J. Martin V. (1978). Anti-acetylcholine receptor antibodies in D-penicillamine-associated myasthenia gravis. *Lancet* 1:1254 10.1016/s0140-6736(78)92481-9 78009

[B157] Viswanath D. V. Jenkins H. J. (1978). Neuromuscular block of the polymyxin group of antibiotics. *J. Pharm. Sci.* 67 1275–1280. 10.1002/jps.2600670922 211223

[B158] Watts J. Brew B. Tisch S. (2015). Myasthenia gravis exacerbation with low dose ocular botulinum toxin for epiphoria. *J. Clin. Neurosci.* 22 1979–1981. 10.1016/j.jocn.2015.05.032 26188667

[B159] Weisman S. J. (1949). Masked myasthenia gravis. *J. Am. Med. Assoc.* 141 917–918. 10.1001/jama.1949.62910130001008 15406884

[B160] Wilson R. W. Ward M. D. Johns T. R. (1974). Corticosteroids: a direct effect at the neuromuscular junction. *Neurology* 24 1091–1095. 10.1212/wnl.24.11.1091 4371087

[B161] Wolfe C. M. Tafuri N. Hatfield K. (2007). Exacerbation of myasthenia gravis during imiquimod treatment. *J. Drugs Dermatol.* 6 745–746. 17763602

[B162] Wolfe G. I. Kaminski H. J. Cutter G. R. (2016). Randomized trial of thymectomy in myasthenia gravis. *N. Engl. J. Med.* 375 2006–2007. 10.1056/NEJMc1611704 27959593

[B163] Wood S. J. Slater C. R. (2001). Safety factor at the neuromuscular junction. *Prog. Neurobiol.* 64 393–429. 10.1016/s0301-0082(00)00055-1 11275359

[B164] Wright J. M. Collier B. (1976). The site of the neuromuscular block produced by polymyxin B and rolitetracycline. *Can. J. Physiol. Pharmacol.* 54 926–936. 10.1139/y76-129 191167

[B165] Xing Q. Zhang Z.-W. Lin Q.-H. Shen L.-H. Wang P.-M. Zhang S. (2020). Myositis-myasthenia gravis overlap syndrome complicated with myasthenia crisis and myocarditis associated with anti-programmed cell death-1 (sintilimab) therapy for lung adenocarcinoma. *Ann. Transl. Med.* 8:250. 10.21037/atm.2020.01.79 32309397 PMC7154453

[B166] Yaari Y. Pincus J. H. Argov Z. (1977). Depression of synaptic transmission by diphenylhydantoin. *Ann. Neurol.* 1 334–338. 10.1002/ana.410010405 31132

[B167] Yeh T. M. Tami J. A. Krolick K. A. (1992). Exacerbated muscle dysfunction by procainamide in rats with experimental myasthenia gravis. *Drug Chem. Toxicol.* 15 53–65. 10.3109/01480549209035172 1555523

[B168] Youssef S. Stüve O. Patarroyo J. C. Ruiz P. J. Radosevich J. L. Hur E. M. (2002). The HMG-CoA reductase inhibitor, atorvastatin, promotes a Th2 bias and reverses paralysis in central nervous system autoimmune disease. *Nature* 420 78–84. 10.1038/nature01158 12422218

[B169] Zeiser R. Blazar B. R. (2017). Pathophysiology of chronic graft-versus-host disease and therapeutic targets. *N. Engl. J. Med.* 377 2565–2579. 10.1056/NEJMra1703472 29281578

[B170] Zeiser R. Blazar B. R. (2018). Acute graft-versus-host disease. *N. Engl. J. Med.* 378 586–586. 10.1056/NEJMc1716969 29414273

